# Ecological and morphological determinants of evolutionary diversification in Darwin's finches and their relatives

**DOI:** 10.1002/ece3.6994

**Published:** 2020-11-10

**Authors:** Ashley M. Reaney, Yanis Bouchenak‐Khelladi, Joseph A. Tobias, Arkhat Abzhanov

**Affiliations:** ^1^ Science and Solutions for a Changing Planet DTP Department of Life Sciences Imperial College London Ascot UK; ^2^ Natural History Museum London UK; ^3^ University de Bourgogne UMR Biogeosciences 6282 CNRS UBFC Dijon France; ^4^ Department of Life Sciences Imperial College London Ascot UK

**Keywords:** adaptive radiation, Darwin's finches, diversification, morphological evolution, seedeaters, tanagers

## Abstract

Darwin's finches are a classic example of adaptive radiation, a process by which multiple ecologically distinct species rapidly evolve from a single ancestor. Such evolutionary diversification is typically explained by adaptation to new ecological opportunities. However, the ecological diversification of Darwin's finches following their dispersal to Galápagos was not matched on the same archipelago by other lineages of colonizing land birds, which diversified very little in terms of both species number and morphology. To better understand the causes underlying the extraordinary variation in Darwin's finches, we analyze the evolutionary dynamics of speciation and trait diversification in Thraupidae, including Coerebinae (Darwin's finches and relatives) and, their closely related clade, Sporophilinae. For all traits, we observe an early pulse of speciation and morphological diversification followed by prolonged periods of slower steady‐state rates of change. The primary exception is the apparent recent increase in diversification rate in Darwin's finches coupled with highly variable beak morphology, a potential key factor explaining this adaptive radiation. Our observations illustrate how the exploitation of ecological opportunity by contrasting means can produce clades with similarly high diversification rate yet strikingly different degrees of ecological and morphological differentiation.

## Graphical abstract

The ecological diversification of Darwin's finches is unmatched on the same archipelago by any other clade of colonising land birds. For all morphological traits, we observe an early pulse of diversification followed by prolonged periods of slower steady‐state rates of change for the tanager family a substantial increase in speciation rate in Darwin's finches coupled with highly variable beak morphology. Our observations illustrate how the exploitation of ecological opportunity by contrasting means can produce clades high in speciation rates yet strikingly different in their degree of ecological and morphological differentiation.

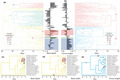

## 1 INTRODUCTION

Our planet's biodiversity is the result of countless evolutionary radiations across a wide range of temporal, geographical, and taxonomic scales. The classic concept of an adaptive radiation involves high levels of ecological diversity evolving rapidly within a single lineage (Losos & Mahler, 2010; Schluter, 2000a), with examples reported from numerous taxonomic groups (Alfaro et al., 2009; Bouchenak‐Khelladi et al., 2015; Moen & Morlon, 2014; O'Leary & et al., 2013; Rabosky et al., 2013). The prevailing view is that these eye‐catching radiations are generated by diversifying selection associated with increased levels of ecological opportunity (Yoder et al., 2010), which can be brought about by key innovations, dispersal to new habitat, or the extinction of competing species (Simpson, 1944, 1953). Both the quantity of available niche space and the size of the initial population are proposed to determine the number and rate of speciation events during a radiation (Gavrilets & Vose, 2005; Ricklefs, 2010), yet they are of little help in revealing the mechanisms which can explain how rapidly and efficiently adaptive radiation takes place. Over the years, evolutionary radiations and their resulting phylogenetic imbalances have attracted multiple explanations ranging from ecology (Rundle & Nosil, 2005; Vrba, 1992), sexual selection (Barraclough et al., 1995; Seddon et al., 2008), co‐diversification with interacting organisms (Farrell, 1998; Moreau et al., 2006), and a propensity to disperse and colonize new areas (Clegg et al., 2002; Moyle et al., 2009).

Simpson (1953) proposed that lineages exploit new ecological opportunities in three ways, or “axes”: geographically, ecologically and evolutionarily. To capitalize on ecological opportunity, a lineage would be expected to undergo evolutionary change whenever directional selection favors individual organisms with particular traits. For example, dispersal to a new island provides access to underexploited resources, thus allowing any colonizing lineage to exploit vacant niche space, provided it has the evolutionary ability to do so (Losos & Ricklefs, 2009). Caribbean *Anolis* lizards (Losos, 2009) and Darwin's finches are classic examples of lineages which have evolved a variety of striking phenotypes and diversified into multiple ecological niches, with the latter having diverged primarily in beak shape and size since their arrival to Galápagos (Grant & Grant, 2008; Sari & Bollmer, 2018; Valente et al., 2015). However, while much is known about avian ecomorphology in relation to phylogenetic history (Pigot et al., 2020), the underlying factors generating the exceptional morphological diversity in Darwin's finches and related Coerebinae remain poorly understood (Mallarino et al., 2012).

Beak morphology in Darwin's finches is incredibly varied, particularly for a clade of its size (see Grant & Grant, 2006). It ranges from the small, thin, and pointed beak of the Green Warbler‐Finch (*Certhidea olivacea*), to the deep, bulky beak of the Large Ground Finch (*Geospiza* *magnirostris*) (Sakamoto et al., 2019). Interspecific competition for limited food resources can drive divergent selection on the beaks of Darwin's finches via character displacement (Grant & Grant, 2006), reducing competition and niche overlap between closely related species. Regardless of the evolutionary mechanisms involved, one of the most intriguing aspects of the Darwin's finch radiation is that it is not replicated in any of the numerous other land birds inhabiting the same archipelago (Grant & Grant, 2008; Valente et al., 2015). For example, the Little Vermilion Flycatcher (*Pyrocephalus *[*rubinus*] *nanus)* and the Yellow Warbler (*Setophaga petechia aureola*) have undergone minimal differentiation compared to their South American mainland or Caribbean counterparts (Valente et al., 2015). The only other avian colonization of the Galápagos to yield multiple species is the Galápagos mockingbirds which appear to have arrived earlier than Darwin's finches yet only diversified into four allospecies with minor ecological and morphological differentiation (Sari & Bollmer, 2018; Valente et al., 2015). On mainland South America, the sister clade of Coerebinae—namely, the Sporophilinae (seedeaters)—has diversified into numerous lineages with rather homogenous beak morphology, presumably via dispersal to new geographic areas rather than dietary divergence (Campagna et al., 2012; Lijtmaer et al., 2004). These two clades illustrate how lineage diversification can proceed by different pathways, only some of which generate high ecological and/or morphological diversity.

Here we use phylogenetic models to explore the contrasting evolutionary radiations of Coerebinae and Sporophilinae and assess the disparity of Darwin's finches compared to other co‐distributed taxa. We evaluate whether the macroevolutionary dynamics of trait evolution among the largely insular Coerebinae are decoupled from those of their continental sister clade of Sporophilinae. To examine the dynamics of trait evolution between and within these taxa, we (a) locate radiating clades within Thraupidae, (b) identify the mode of trait evolution during their evolutionary history, and (c) explore morphological disparity among clades. We predict that shifts in morphological trait evolution will be more evident in Darwin's finches than most of their Caribbean relatives and the rest of Thraupidae. We also expect to find more shifts specifically in the evolution of beak traits, compared to body traits, and for those shifts to delineate distinct subclades within Thraupidae.

Following this investigation, we directly compare beak shape diversity in Darwin's finches, mockingbirds, and other Galápagos bird endemics to examine how beak traits have diversified in different lineages colonizing the same environment. Based on observed variation in beak shape across these radiations, we test whether Darwin's finches evolved to occupy a larger area of morphological space compared to other endemic taxa in relation to their respective parent lineages. We also assessed the role of ecological selection by examining whether the extent, kind, and directionality of clade‐specific beak shape variation are reflected in dietary niches.

## 2 METHODS

### 2.1 Trait data

Much of the data relevant to this study was published by Drury et al. (2018) but we include data from an additional 30 species and add two morphological traits (one beak length measure and hand‐wing index) described in the supplementary material. We collected morphological trait data for all 349 species of Thraupidae presented in the maximum clade credibility (MCC) tree from Burns et al. (2014). Measurement methods are described in detail elsewhere (Drury et al., 2018; Trisos et al., 2014) and summarized briefly here. We aimed to measure at least four (average = 5.7) museum specimens to compile data on eight continuous linear morphological measurements (given to the nearest tenth of a millimeter): culmen length, beak tip to anterior edge of nostrils, beak width and depth, tarsus length, wing length (from the bend of the wing to the tip of the longest primary), and tail length (from the tip of the longest rectrix to the point at which the two central rectrices protrude from the skin). We took the difference between wing length and first secondary length to calculate hand‐wing index, a widely used index of dispersal ability and flight efficiency in birds (Claramunt et al., 2012; Pigot & Tobias, 2015). For all study species, we compiled body mass (in grams) from data aggregated by Wilman et al. (2014) and all morphological data were log‐transformed prior to conducting analyses. Extracted primary diet classification of each species was taken from Tobias and Pigot (2019) which uses the procedure described by Felice et al., (2019) to reclassify proportional diet data in Wilman et al. (2014) into one of six food types (invertebrates, carrion, fruit, nectar, seeds, and plants).

The clade of Darwin's finches studied here consists of 14 genetically and morphologically distinct species. Recent phylogenomic studies have treated up to four additional Darwin's finch taxa as species (Lamichhaney et al., 2015, 2016), all of which are distinct genetically but not morphologically. We were not able to include these forms in our study as they are absent from phylogenies spanning the entire tanager clade (e.g., Burns et al. (2014)) and therefore incompatible with our comparative trait analyses.

### 2.2 Phylogenetic data and taxonomic diversification regimes

The latest available MCC tree of Thraupidae was obtained from Burns et al. (2014). It contains 349 (93%) of the 377 tanager species listed by Clements et al. (Clements et al., 2019). To quantify diversification rates across the tree, we following Burns et al. (2014) in using the Bayesian Analysis of Macroevolutionary Mixtures (BAMM) program (Rabosky, 2014), which is designed to detect and quantify heterogeneity in evolutionary rates using a reversible‐jump Markov chain Monte Carlo (rjMCMC). Although recent criticisms have drawn attention to weaknesses in BAMM estimation of diversification rates and rate shifts (Meyer et al., 2018; Meyer & Wiens, 2017; Moore et al., 2016), the package matches or outperforms other methods when supplied with equivalent information (Rabosky, 2018; Rabosky et al., 2017). We do not attempt to infer speciation rates, but rather the net result of speciation minus extinction, that is, diversification.

We ran two MCMCs for 100,000,000 generations sampling every 1,000 generations, checking for convergence by plotting the log‐likelihood trace of the MCMC output file and ensuring effective sample sizes exceeded 200 (after 30% burn‐in) using the “coda” package (Plummer et al., 2006). A single expected shift, typical of trees with <500 tips, was computed following configuration of the control file according to BAMM specifications (http://bamm‐project.org/quickstart.html). Using the “BAMMtools” package in R (Rabosky, 2014), we identified the maximum a posteriori probability (MAP) shift configuration (the distinct shift configuration with the highest posterior probability). Similar methods were previously applied to the tanager family by Mason et al. (2017).

### 2.3 Shifts in trait diversification regimes

To describe the statistical patterns in the data inferred from both trait data and the MCC tree, we used the reversible‐jump algorithm that allows the identification of regimes present in phylogenetic comparative data without a priori hypotheses. The “bayou” package jointly estimates the location, number, and magnitude of shifts in adaptive optima (Uyeda & Harmon, 2014). We created a prior that allowed for any number of shifts per branch with the probability proportional to their branch length. Two runs of 2,000,000 iterations were performed to check for convergence.

Regimes for all branches in the phylogeny were then defined based on those identified from the bayou analyses for each trait. The “OUwie” package fits OU‐based models whereby traits evolve under discrete selective regimes and the models themselves are allow to vary in given parameters (Beaulieu et al., 2012). Under an OU process, trait values evolve toward an optimum (θ), which can be a single value for all lineages or can vary among the predefined regimes. For each model, the rate of stochastic evolution (σ^2^) and the strength of directed evolution toward the optima (α) can be set to be equal across all regimes but with different phylogenetic state means (“OUM”) or to differ between regimes (“OUMA” where only α can vary; “OUMV” where only σ^2^ can vary; “OUMVA” where both α and σ^2^ can vary). To select the optimal model, we used the second order Akaike information criterion (AICc), taking into account sample sizes and a penalty increase for model complexity.

These methods (using “OUwie,” “bayou,” and “pmc” packages (Boettiger et al., 2012)) were applied to the Coerebinae and Sporophilinae clades and their closest outgroup, a clade containing subfamilies Saltatorinae, Emberizoidinae, and Poospizinae (Burns et al., 2014). This was performed to (a) clarify patterns of character evolution among the closet relatives of our two focal clades and (b) understand how those patterns of evolution vary between and within the focal clades. To illustrate variation in the mode of trait evolution, we plotted trait histograms against phylogenetic trees with posterior probabilities produced via the trait diversification analysis.

### 2.4 Trait disparity among dietary guilds

Since the beak morphology of species with identical or similar dietary compositions may be correlated, the independence assumptions made under traditional regression approaches or mixed models may be violated. Generalized estimation equation (GEE) addresses this problem by describing changes in a mean (in our case, beak measurements) given changes in covariates (in our case, diet) accounting for nonindependence (Hubbard et al. 2010). We implemented a GEE model using the “ape” package (Paradis & Claude, 2002) to assess whether observed variation in beak morphology and overall morphology are associated with species diet. The model uses a correlation matrix specifying whether the dependence among observations are discrete or continuous (Paradis & Claude, 2002). We modeled the four linear beak dimensions against the primary diet category for all thraupid species under the approximation that the distribution of events in the analysis was binomial. Beak measurements were included as predictors and diet was treated as a binary response since species were assigned to a particular dietary category if it made up over 50% of their dietary composition (see trait data above).

To obtain a Euclidean representation of the noncontinuous dietary information, a symmetric similarity/distance matrix was calculated from the original matrix of 353 species and 6 dietary items to conduct principal coordinates analysis (PCoA) in the “vegan” package (Oksanen et al. 2019).

## 3 RESULTS

### 3.1 Diversification rates and rate shifts

**FIGURE 1:**
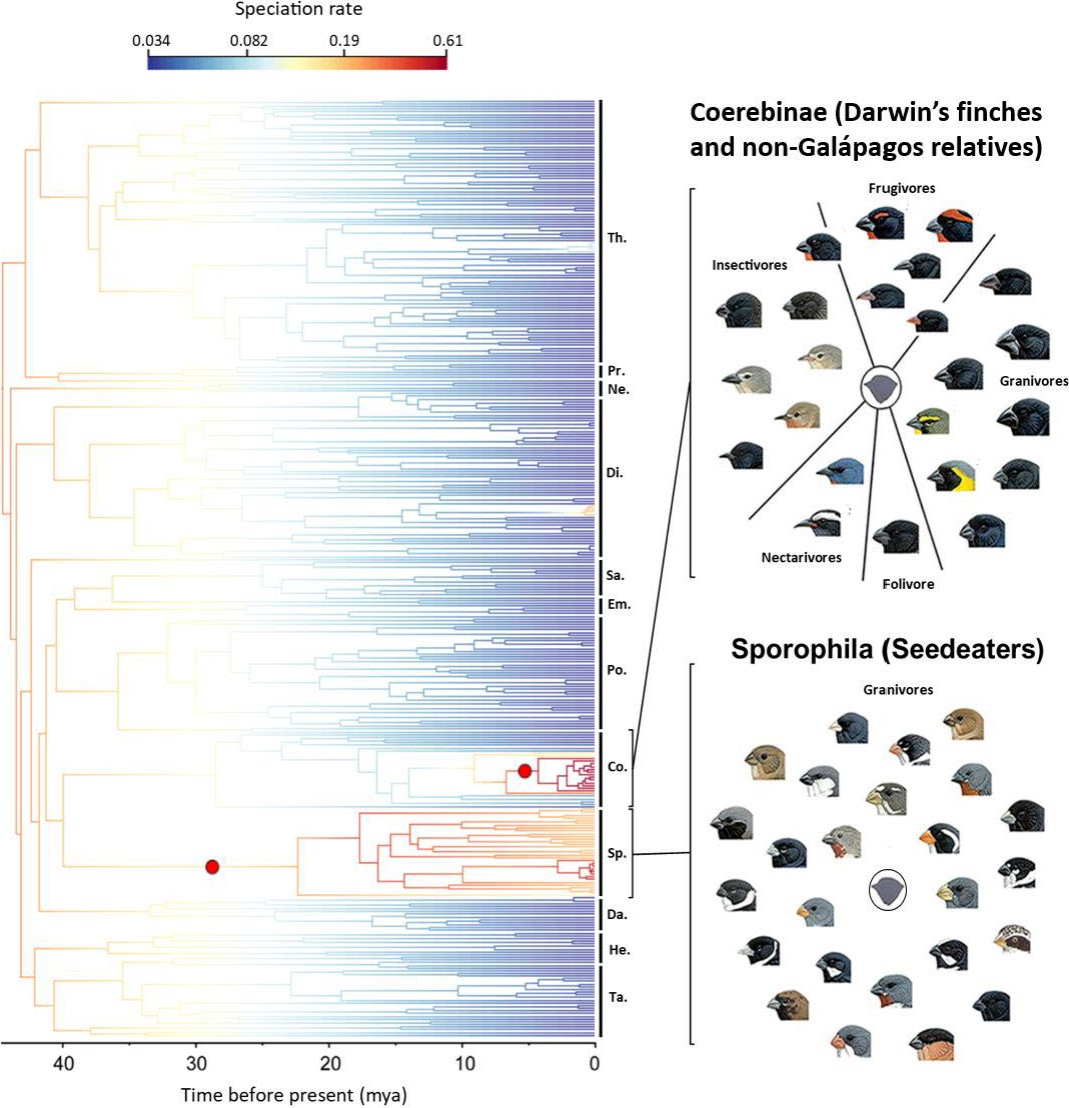
Maximum clade credibility (MCC) Thraupidae timetree showing rate‐shifts in diversification estimated from BAMM analysis. Red‐filled circles indicate locations of shift configurations in net diversification rate. The coloured section of each branch represents the mean of the posterior density of diversification rate with blue and red areas indicating low and high diversification rates respectively. The abbreviations of Thraupidae subfamilies are given to the right of the tree (Th, Thraupinae; Pr, Porphyrospizinae; Ne, Nemosiinae; Di, Diglossinae; Sa, Saltatorinae; Em, Emberizoidinae; Po, Poospizinae; Co, Coerebinae; Sp, Sporophilinae; Da, Dacninae; He, Hemithraupinae; Ta, Tachyphoninae). The monotypic/bitypic subfamilies Charitospizinae, Catamblyrhynchinae and Orchesticinae are included in the tree but not labelled. Images are reproduced with permission from Cornell Lab of Ornithology

Our BAMM results reveal a general pattern of decreasing rates over time in Thraupidae (shift from red to blue along phylogenetic branches) with the Darwin's finch and Sporophilinae clades being two notable exceptions undergoing significantly higher diversification rates. The MAP shifts found in BAMM support this conclusion, with one shift present in the Darwin's finch clade on the branch after the appearance of the Green Warbler‐Finch (*Certhidea olivacea*) and a second at the base of the crown Sporophilinae clade (Figure 1). Higher rates of diversification in these two clades were previously reported by Burns et al. (2002), Burns et al. (2014) and Mason et al. (2017), and are also present in the most complete phylogeny of living birds (Jetz et al., 2012). The rate‐through‐time plot further supports an increase in diversification rate for the Coerebinae clade ~ 6 Mya and for Sporophilinae beginning ~ 21 Mya (Figure S1). Within the set of distinct shift configurations sampled during the MCMC (Figure S2), 12% of the samples from the posterior are assigned to a single shift configuration containing a single shift at the bases of the Darwin's finch and Sporophilinae clades. The macroevolutionary cohort matrix shows the majority of Thraupidae are united under a single rate dynamic with strong support for Sporophilinae and Darwin's finches being a separate cohort, both from the Thraupidae family and from each other (Figure S3). Within Sporophilinae, there is weak support for the recent “capuchino” radiation having a unique rate dynamic compared to other clade members.

### 3.2 Shifts in morphological trait regimes

**FIGURE 2:**
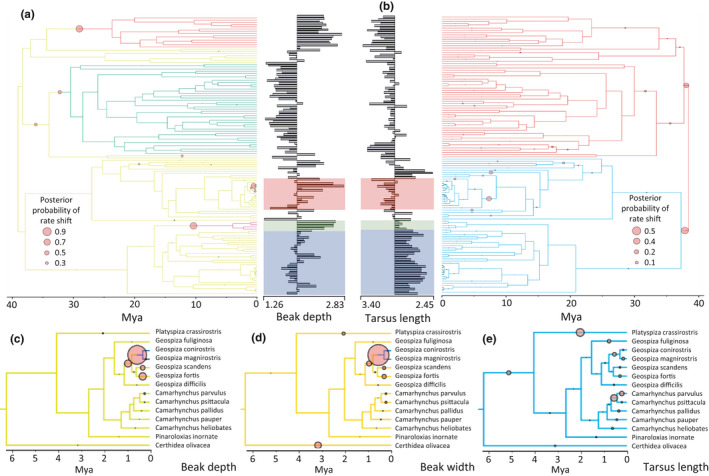
Maximum clade credibility (MCC) Thraupidae timetree from bayou analyses showing trait regimes. Branches with different colours represent taxa with different regimes of a particular trait. Red‐filled circles indicate locations of a shift from one trait regime to another. (a, b) Trait regimes of the subfamilies Saltatorinae, Emberizoidinae, Poospizinae, Coerebinae and Sporophilinae (see Figures S4–S13 for fully labelled tree images). Shaded rectangles represent the clades Darwin's finches (red), Oryzoborus (green) and Sporophila (blue). Trait histograms of log transformed trait values above and below the trait mean are shown next to the phylogenies. (c–e) Close‐up of Darwin's finch phylogenies comparing regime shifts for beak and non‐beak related traits. Posterior probabilities of the regime shift in panels c and d are 0.73 and 0.81, respectively. Axes below phylogenies indicate millions of years before present (mya)

Bayou analysis detected three locations on the tree where large beak depth increases, namely in members of *Saltator*, ground finches (*Geospiza* spp.), and seed finches *Oryzoborus* (Sporophilinae) (Figure 2). Tarsus length is more variable across the Thraupidae phylogeny, with outgroups generally possessing longer tarsi and Darwin's finches and seedeaters evolving relatively short tarsi (Figure 2). Larger body mass has evolved in *Saltator* and *Oryzoborus*, but small body mass is conserved among the rest of Sporophilinae. Changes in beak width closely matched the regime shifts observed for beak depth, with the large Caribbean bullfinches (*Loxigilla* spp. and *Melanopyrrha* spp.) evolving wider beaks than other Coerebinae members. Significant shifts in beak width are also detected in several species of Darwin's finches (*Geospiza* spp.) and in seed finches (*Oryzoborus* spp.). At least four shifts in total beak length have occurred during evolution of Thraupidae: Sporophilinae has evolved shorter beaks, while *Oryzoborus*, *Saltator*, *Geospiza magnirostris,* and *G. conirostris* have all evolved shorter and deeper beaks.

A minority of the shifts we identified in beak trait evolution occurred on branches descending from nodes with low posterior probabilities among Coerebinae (e.g., beak width and depth in *Geospiza magnirostris* and *G. conirostris*). A more recent phylogeny of Darwin's finches (Lamichhaney et al., 2015) provides full local support for the sister relationship of these taxa, suggesting that our interpretations are not affected by inaccurate or poorly supported nodes. For shifts in the evolution of other morphological traits, see Figures S4–23, Tables S1 and S2 for posterior probabilities of nodes, and Table S3 for specific shifts in trait regimes and Table S4 for the parameter values for all traits analyzed across Thraupidae taxa.

Fitting BM and OU models of trait evolution to beak length variation across Thraupidae revealed the best fitting model was OUMVA where both the rate of stochastic evolution (σ^2^) and the strength of directed evolution toward the optima (α) are allowed to vary. The AICc values for the OUMVA model were much lower than those for other models tested, with values for the simpler OU models (OU1, OUM, OUMV) similar to those under BM models (Table 1). Applying BM models across the phylogeny using stochastic evolution recognizes a high number of regimes or measurements that differ significantly between lineages. Model fits and parameter estimates for the remaining eight morphological traits are given in the supplementary material (Table S5). The OUMVA model was again the best fitting model, followed by the weaker OUMA model, which in turn had better fit than simpler OU and BM models when analyzed for beak depth, beak width, wing length, and tail length (Table S5).

**TABLE 1:** Comparison of “OUwie” model parameters of beak length (culmen) across the Thraupidae phylogeny

Variable	BM1	BMS	OU1	OUM	OUMV	OUMA	OUMVA
No. of shifts	7	7	7	7	7	7	**7**
Loglik	−823.93	−763.45	−764.56	−701.34	−675.53	5,764.29	**2.05 × 10^9^**
Half‐life	‐	‐	10.95	2.66	2.74	1.21	**5.61**
AICc	1651.89	1,560.52	1535.18	1,423.33	1,386.88	−11492.76	**−4.09 × 10^9^**
Param.count	2	16	3	10	17	17	**24**

Note

Loglik, Log‐likelihood values taken from the “OUwie” package. Bold values denote the best fitting model. See Table S5 for OUwie model comparisons for other morphological traits.

### 3.3 Dietary analysis and feeding guilds

**FIGURE 3:**
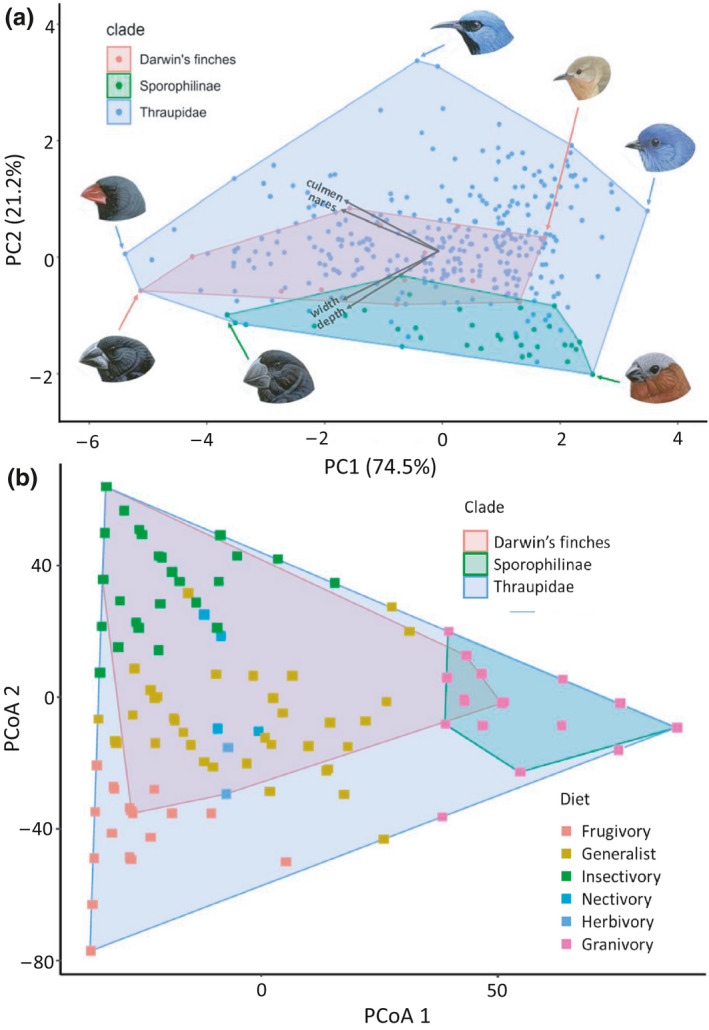
(a) Principal component analysis (PCA) morphospace of the four beak traits for Thraupidae, Sporophilinae and Coerebinae clades. Species at the extremes of their respective clades are shown around the outside of the morphospace. Species heads are, clockwise from the top, Cyanerpes lucidus, Certhidea olivacea, Xenodacnis parina, Sporophila cinnamomea, Oryzoborus atrirostris, Geospiza magnirostris and Saltator fuliginosus. (b) PCoA plot of dietary composition values of Thraupidae and subclades based on Euclidean distances of dietary composition values. Points correspond to the major dietary component (i.e. the food‐type constituting 60% or more of species' diet) with areas shaded according to taxonomic clade. Images are reproduced with permission from Cornell Lab of Ornithology

All 36 members of Sporophilinae (seedeaters) are tightly clustered with other granivores in the PCoA diet space, whereas the 14 Darwin's finch species cover the majority of the diet space occupied by the 349 tanager species representing the entire Thraupidae family (Figure 3b). This area of occupied diet space is expanded further when Darwin's finches are combined with their Caribbean relatives (Figure S24) owing to the inclusion of the nectivorous *Coereba flaveola*, nectivorous/frugivorous *Euneornis caqmpestris,* and the granivorous specialists of *Tiaris spp*. as shown by Burns et al. (Burns et al., 2002).

The results of the GEE analysis show there are significant differences in beak morphology among the various dietary guilds (Table S6). For example, each 1‐unit increase in culmen length and beak depth is associated with 0.9 increase and 0.4 decrease in invertebrate diet, respectively. These slopes suggest a significant inverse association between these beak dimensions in insectivores, which is expected as they typically have relatively long and thin beaks.

## 4 DISCUSSION

### 4.1 Morphological evolution: Coerebinae versus Sporophilinae

Coerebinae and Sporophilinae are related clades with significantly higher net diversification rates compared to other tanagers. However, both clades show distinct patterns of morphological evolution relating to beak and body size. Numerous unique selective regimes for various aspects of beak morphology and body mass occur throughout Coerebinae and in the lineage leading toward the four *Oryzoborus* species (Sporophilinae). This implies that selection acts on multiple trait axes, sometimes driving species toward new adaptive optima for those respective traits (see methods). These shifts in body mass and beak morphology accurately delineate subclades within Coerebinae and Sporophilinae. Shifts in beak depth and width are notable in the Coerebinae bullfinches of the Caribbean and particularly in two species of Darwin's finches: *Geospiza magnirostris* and *G. conirostris*. In the latter cases, the pattern of morphological change is consistent with some other examples of species‐rich avian clades with high disparity and diversity of beak morphology (Burns et al., 2002; Lovette et al., 2002; Price, 2008). This is particularly striking considering that other morphological traits of Darwin's Finches, such as tarsus, wing, and tail length, have either not undergone regime shifts or else show regime shifts that do not correspond to any major taxonomic units.

The high number of shifts in beak evolution detected among the internal branches of Darwin's finches may be biased by incomplete sampling, but this seems unlikely to be the case since the species missing from our phylogenetic analysis, including the Grey Warbler‐Finch (*Certhidea fusca*) and the Vampire Finch (*Geospiza septentrionalis*), are morphologically similar to their sister species (*C. olivacea* and *G. difficilis*, respectively). They lack extreme beak morphologies and fall well within the bounds of the morphospace occupied by Darwin's finches as a group (Tokita et al., 2017). Our analyses are therefore unlikely to have overlooked any regime shifts in beak morphology (akin to the beak depth and width shifts for *G. magnirostris and G. conirostris*).

Our findings confirm that large‐scale shifts in morphology have generally occurred early in Thraupidae evolution with fewer and smaller changes in all measured traits occurring toward tips of the phylogeny. Similarly, we find evidence of early regime shifts for entire clades (subfamilies and genera), after which the tempo of evolutionary change becomes stable with mostly minor fluctuations in recent times (Figure 2 and Figures S4–S23). Although we are not able to evaluate the morphological diversity of extinct taxa, our findings are congruent with the theory that extant organisms showed maximum morphological variation early in their evolutionary histories (Erwin, 2007; Foote, 1997; Hughes et al., 2013; Ruta et al., 2006). We also uncover patterns consistent with recent studies of beak evolution showing that early diversification of beak traits subsequently transitioned to comparatively stable rates over time, both viewed across birds in general (Cooney et al., 2017), and when focusing exclusively on divergence in closely related species (McEntee et al., 2018). This process is sometimes referred to as packing (Cooney et al., 2017; Pigot et al., 2016), whereby higher taxon‐level variation is produced early and then filled (or packed) by subsequent lineages. There are two major exceptions to this trend in Thraupidae: the entire Coerebinae clade and Sporophilinae, which both recently underwent a pulse of lineage diversification according to phylogenetic data. This was coupled with morphological diversification only in Coerebinae.

What factors can help explain the observed patterns of morphological evolution in the tanager family? External factors such as available niches and natural selection are undoubtedly important and provide the usual explanation for early bursts of avian morphological evolution, since these can arise from rapid saturation of ecological niches and consequent narrowing of ecological opportunity (Price et al., 2014; Tobias et al., 2020). On the other hand, it may also be interpreted as evidence of relatively greater flexibility of developmental programs early in evolution and subsequent canalization and integration of genetic mechanisms which begin to constrain the available variation in each clade (Abzhanov, 2017). Indeed, a range of intrinsic factors may play a pivotal role because the “evolvability” of a particular lineage can be increased through purely genetic mechanisms—such as mutation, or admixture between populations—as well as changes in developmental programs underlying morphological traits (Gillespie et al., 2020; Payne & Wagner, 2019; Tobias et al., 2020). Coerebinae appears to be the only group of tanagers which deviate from the trend of early bursts by producing significant variation in beak morphology later in their evolution, perhaps reflecting changes in the craniofacial developmental program that increased their evolutionary variability. In all cases, new morphological traits may be generated by preceding changes in development, such that greater morphological diversity implies a higher degree of alteration to the underlying genetic and molecular mechanisms.

### 4.2 Dietary disparity: Coerebinae versus Sporophilinae

The importance of adaptation to ecological niche vacancies is highlighted by the fact that beak diversity reflects a wide range of food types among tanagers, including invertebrates, seeds of various sizes, nectar, fruit (from small berries to large items), leaf buds, and occasionally scavenging and feeding on blood. Compared with their primarily granivorous ancestors, Darwin's finches occupy an extensive and, importantly, central portion of the beak shape morphospace (Figure 3b). This pattern suggests that their radiation on the Galápagos Islands has involved the evolution of beaks along diverging, even opposing dimensions, and that the variety of foraging niches nearly matches that of the entire tanager family. The scale of this radiation of forms contrasts sharply with the much smaller beak morphospace occupied by Sporophilinae, which is largely skewed toward short conical beaks. This more restricted morphospace is reflected in the fact that diets have not diversified extensively in Sporophilinae, being largely restricted to granivory. Whether this is because Sporophilinae have lower intrinsic evolvability, or simply because they are presented with fewer ecological opportunities in the “crowded marketplace” of continental assemblages, is presently unclear (Day et al., 2020).

### 4.3 Comparison of passerine radiations on Galápagos

**FIGURE 4:**
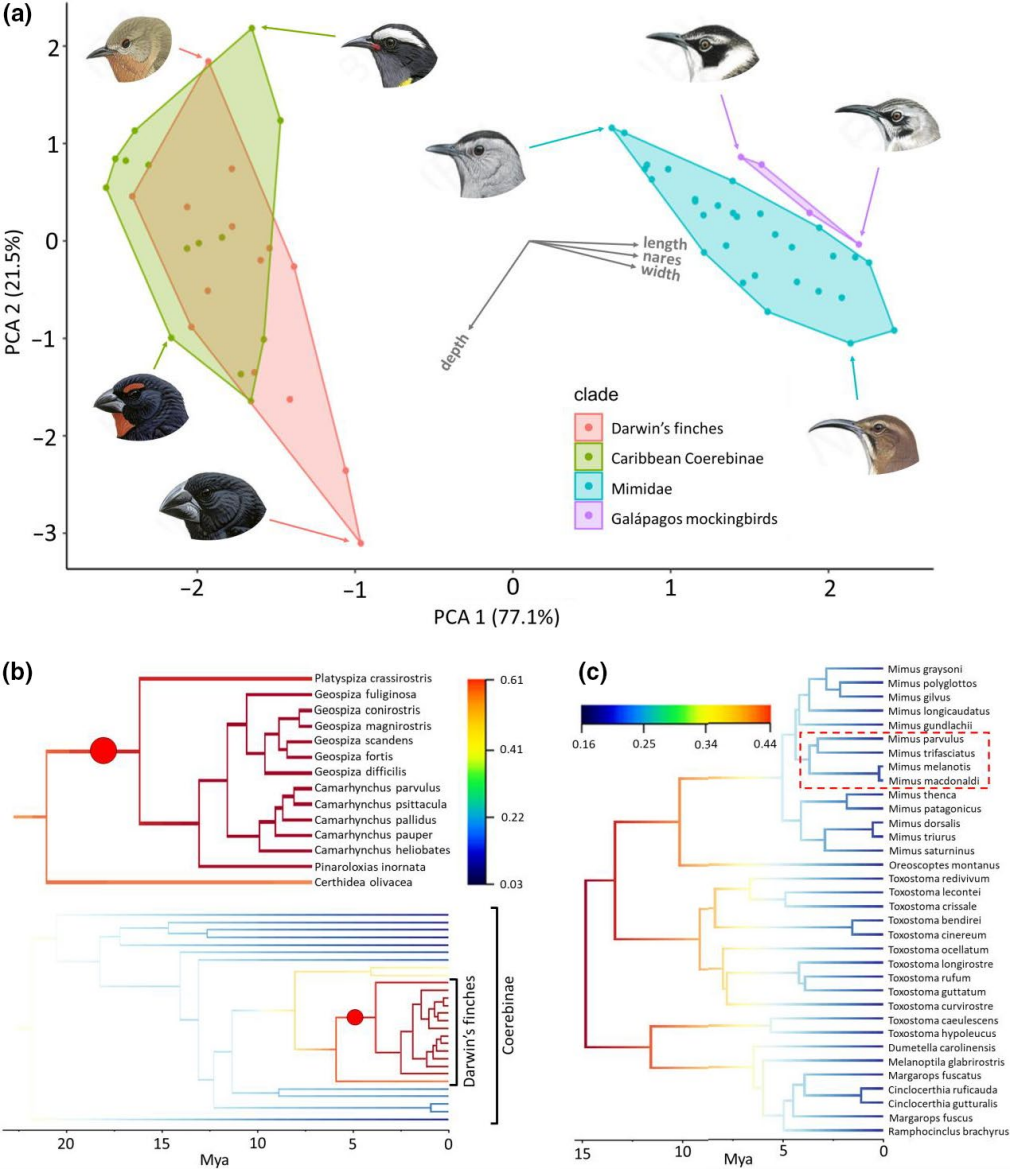
Comparison of speciation rate and beak morphology of Darwin's finches, the Galápagos mockingbirds and their respective families/subfamilies. (a) Beak morphospace of Darwin's finches, Caribbean Coerebinae (including mainland South American forms), Galápagos mockingbirds and Mimidae (including both Caribbean and mainland forms). Loadings of the first two components are given as labelled arrows and explained variance given along the axes. Species heads from left to right, top to bottom are: Certhidea olivacea, Coereba flaveola, Mimus parvulus, Mimus macdonaldi, Dumatella carolinesis, Melanophyra nigra, Toxostoma ocellatum and Geospiza magnirostris. (b) Close‐up of the MCC tree from the BAMM analysis showing diversification rates of Coerebinae and Darwin's finches. Coloured branches and circles are as in Figure 1. (c) Diversification rate of the Mimidae family (tree obtained from www.birdtree.org) from the BAMM analysis with the Galápagos mockingbirds outlined in the red dashed box. Scales in c and d show speciation rates of the respective clades and axes below BAMM trees show time before present (mya). Images are reproduced with permission from Cornell Lab of Ornithology

A single bird species likely resembling extant mainland *Tiaris* grassquits, which specialize on grass seeds, colonized the Galápagos Islands and diversified to produce all modern‐day Darwin's finches (Sato et al., 2001). The scenario suggested by phylogenetic relationships among extant species is that the granivorous grassquit‐like ancestor first gave rise to insectivorous Warbler finches and generalist Cocos finch and Sharp‐billed finches, and then to all other forms (Grant & Grant, 2008). The ancestral lineage was a member of the morphologically diverse Coerebinae clade, and over the course of speciation and diversification in a new geographic setting was able to re‐generate and then surpass the beak diversity of its ancestral clade (red versus yellow‐green polygons on Figure 4a)(Price, 1987; Schluter, 2000b). Note that while phylogenetic data suggest a very recent radiation in *Geospiza,* it is possible that morphological diversification of the clade occurred earlier in its history, with levels of current molecular divergence reduced by repeated rounds of island colonization and introgression (Tobias et al., 2020).

**FIGURE 5:**
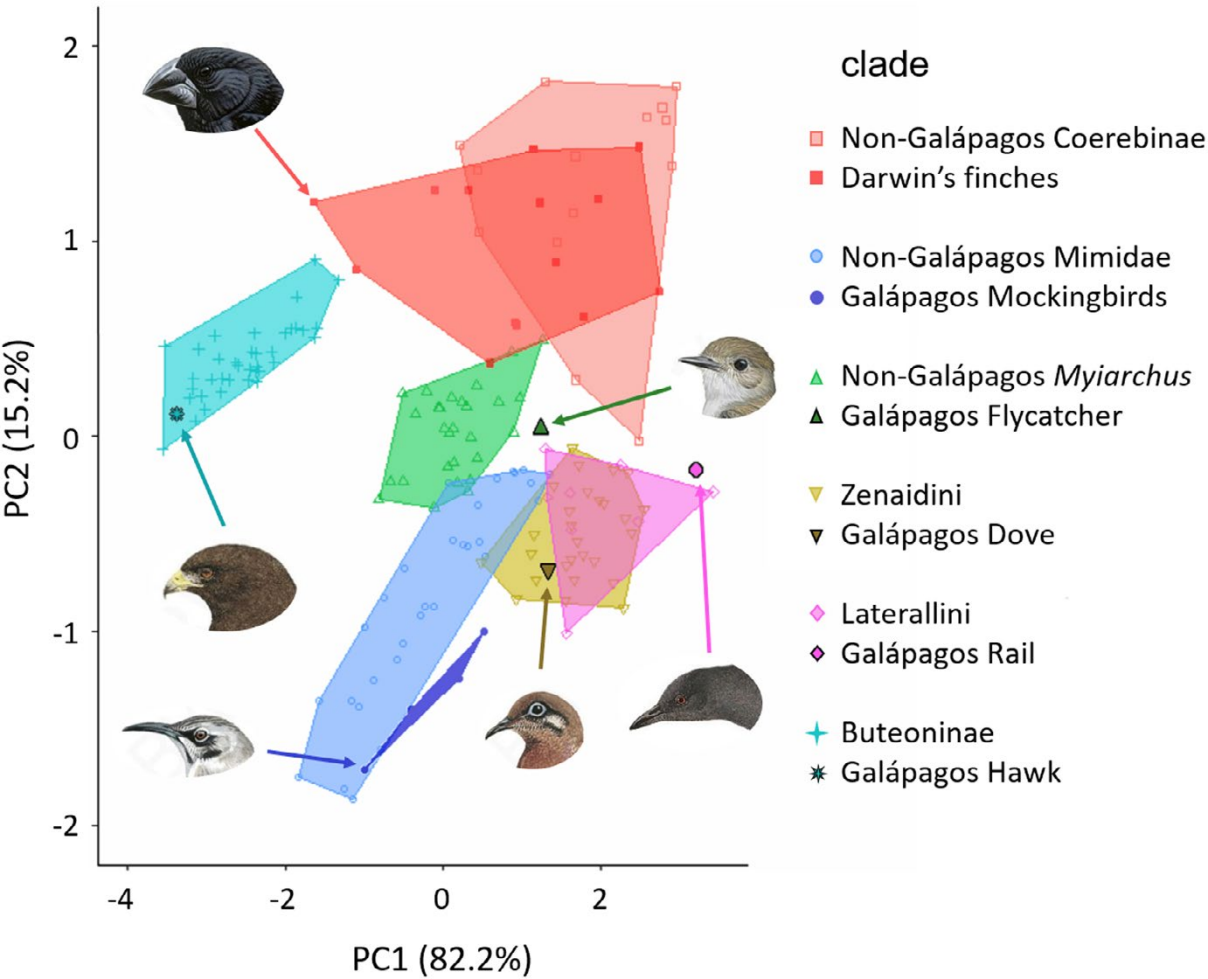
PCA morphospace of beak morphology of endemic Galápagos species and their respective ancestral monophyletic clades. “Non‐Galápagos” Coerebinae, Mimidae and Myiarchus include all Caribbean and continental lineages in those clades. Species illustrated, clockwise top left: Geospiza magnirostris, Myiarchus magnirostris, Laterallus spilonota, Zenaida galapagoensis, Mimus macdonaldi and Buteo galapagoensis. Images are reproduced with permission from Cornell Lab of Ornithology

The Galápagos mockingbirds colonized the islands at a similar time (Table 2), yet subsequently produced only four allopatric and morphologically similar species. They clearly adapted to the ecological conditions of the Galápagos by evolving new beak morphologies outside the ancestral mainland/Caribbean beak morphospace but did not explore it further (pink versus blue polygons on Figure 4a). Likewise, net diversification rates in Galápagos mockingbirds did not change following their dispersal to Galápagos (Figure 4c). All other land birds failed to diversify and expand to new ecological niches after arriving to Galápagos with some invading lineages, such as rails, flycatchers, doves, and hawks, producing single endemic species morphologically similar to their ancestors and relatives on the mainland (Figure 5 and Table 2).

**TABLE 2:** Estimated colonization times of Galápagos avifauna

Lineage name	Galápagos species	Stem age (mya)	Data sources
Darwin's finches	Radiation (14 + species)	3.03 (2.22–3.85)	(Farrington et al., 2014)
Galápagos mockingbirds	Radiation (4 species)	3.96 (3.35–4.55)	(Lovette et al., 2012)
Galápagos flycatcher	*Myiarchus magnirostris*	0.86 (0.58–1.13)	(Sari & Parker, 2012)
Galápagos dove	*Zenaida galapagoensis*	3.51 (2.57–4.65)	(Johnson & Clayton, 2000)
Galápagos rail	*Laterallus spilonota*	9.50 (7.00–14.00)	(Garcia et al., 2014)
Galápagos hawk	*Buteo galapagoensis*	0.13 (0.05–0.25)	(Bollmer et al., 2005)

Note

Ages shown are mean estimates and 95% highest posterior density across posterior distribution of trees from BEAST. Stem ages estimates for Darwin's finches, Galápagos mockingbirds, flycatcher, and dove are taken from Table S1 in Valente et al., 2015. Note that estimates of the timing of colonization based on molecular divergence are sensitive to a number of potential biases including variation in rates of molecular evolution and introgression during more recent waves of colonization (Tobias et al., 2020).

Such differences in the propensity for morphological diversification across clades are often attributed to the underlying developmental mechanisms, more specifically to high cranial modularity, that is, ability of different parts of the cranium to change independently of each other (Bright et al., 2016; Felice & Goswami, 2018; Wagner et al., 2007). For example, it was previously shown that the beak developmental program in Darwin's finches is highly modular potentially allowing for different beak traits, such as beak depth or length, to evolve autonomously (Abzhanov et al., 2004, 2006). This appears to be a genetic trait shared with closely related species of Coerebinae (Abzhanov, 2017; Mallarino et al., 2012).

A related view—complementary rather than contradictory—is that rapid evolution of novel morphologies may be promoted by patterns of integration at the level of the entire cranium, that is, an increased level of developmental connectedness and genetic covariation among cranial elements, particularly when selection favors changes along the line of maximum covariation (Navalón et al., 2020). Specifically, the higher cranial integration of Darwin's finches and Hawaiian honeycreepers has been proposed to explain why they underwent adaptive radiation following dispersal to islands, involving greater changes in beak and skull allometry (Tokita et al., 2017). Our findings are consistent with the hypothesis that changes in developmentally regulated organization of the modular avian cranium specific to Darwin's finches and their close relatives can help to explain why (and how) they have produced higher levels of novel adaptive variation compared to other tanagers and to other Galápagos birds.

Our study explores the patterns of morphological and dietary variation to better understand the relative significance of extrinsic and intrinsic factors in the diversification of Darwin's finches and other similar adaptive radiations. It has long been known that the ancestor of the Coerebinae gave rise to a multitude of morphologically and ecologically distinct forms both on the Caribbean and Galápagos archipelagos, and our analyses confirm that other avian colonists to the Galápagos have not undergone such dramatic modifications. We argue that the ability of Coerebinae to generate multiple new ecomorphs resulted partly from intrinsic factors, perhaps including increased flexibility in their craniofacial developmental program which probably predated their expansion to Galápagos. Nonetheless, environment‐driven factors, such as ecological constraints and purifying selection imposed by available trophic niches, must have played an important role. In particular, a narrow arena of ecological opportunity can help to explain low rates of diversification among ecological specialists in Coerebinae, including Bananaquit (*Coereba flaveola*), Vegetarian Finch (*Platyspiza crassirostris*), and warbler finches. The range of morphological diversity in Coerebinae therefore results from an interplay between intrinsic and extrinsic factors, with some lineages apparently shaped more by evolvability and others by ecology.

## 5 CONCLUSIONS

Our finding that two closely related clades of tanagers (Coerebinae and Sporophilinae) have undergone high rates of net diversification despite markedly different evolutionary histories in contrasting geographical settings suggests that rapid morphological diversification is not necessarily coupled with a particular mode of speciation. The most obvious difference is that Coerebinae speciation is associated with rapid changes in beak morphology linked to partitioning of ecological resources. Members of Sporophilinae have experienced higher net diversification rates with much lower morphological diversification. The Darwin's finches occupy a far larger area of the beak morphospace relative to that of other Galápagos endemic clades evolving novel beak morphologies in addition to forms similar to those of their Caribbean relatives. While ecological and biogeographic factors no doubt play an important role, our findings support the hypothesis that the ancestor of Darwin's finches arrived on the Galápagos Islands already endowed with the genetic propensity to produce the high levels of beak variation needed to explore new dietary niches. A similar combination of increased developmental variability and availability of new ecological opportunities may apply to other adaptive radiations in passerine birds (e.g., Hawaiian honeycreepers, Malagasy vangas). Future research should focus on the evolution of developmental genetic programs, including that controlling beak morphology, ideally comparing both quickly diversifying and morphologically conservative clades, to determine how changes in genetic mechanisms contribute to avian diversification.

## CONFLICT OF INTEREST

The authors declare no competing interests.

## AUTHOR CONTRIBUTION

**Ashley Reaney:** Conceptualization (equal); Data curation (equal); Formal analysis (equal); Investigation (equal); Methodology (equal); Visualization (equal); Writing‐original draft (lead); Writing‐review & editing (equal). **Yanis Bouchenak‐Khelladi:** Conceptualization (equal); Formal analysis (equal); Methodology (equal); Validation (equal); Visualization (equal); Writing‐review & editing (equal). **Joseph Andrew Tobias:** Conceptualization (equal); Data curation (equal); Investigation (equal); Methodology (equal); Validation (equal); Visualization (equal); Writing‐review & editing (equal). **Arkhat Abzhanov:** Conceptualization (equal); Funding acquisition (equal); Investigation (equal); Methodology (equal); Project administration (equal); Resources (equal); Supervision (lead); Validation (equal); Visualization (equal); Writing‐original draft (equal); Writing‐review & editing (equal).

## Supporting information

App S1

Data S2

App S3

Data S4

## Data Availability

The authors declare that all data supporting the findings of this study are available in the Appendix of this paper or on the figshare online repository. Morphological data available at https://figshare.com/s/0d1be17c369f65266598. PCA values used for analyses available at https://figshare.com/s/5512a06b8525f26aeadf
