# Supplementary material for: Ecological and morphological determinants of evolutionary diversification in Darwin's finches and their relatives
Source: Ecol Evol. 2020 Nov 10;10(24):14020–32. doi: 10.1002/ece3.6994 (PMC7771120; doi:10.1002/ece3.6994)
Supplement: Supplementary file 1 — App S1 [file ECE3-10-14020-s001.docx]

**APPENDIX S1**

**List of supporting figures and tables**

**Figure 1** Speciation and extinction rates through time of Sporophilinae, Coerebinae and Thraupidae

**Figure 2** Distinct shift configurations within the Thraupidae phylogeny

**Figure 3** Macroevolutionary cohort matrix of the family Thraupidae

**Figure 4-13** Bayou trait simmaps of Coerebinae and outgroups

**Figure 14-23** Bayou trait simmaps of Thraupidae

**Figure 24** PCoA plot of diet composition of Thraupidae, Coerebinae and Sporophilinae

**Figure 25** Contributions of beak traits to principal component axes in fig. 4a

**Figure 26** Contributions of beak traits to principal component axes in fig. 5

**Table 1** Posterior probabilities of Darwin’s finch nodes in the MCC tree

**Table 2** Posterior probabilities of Sporophilinae nodes in the MCC tree

**Table 3** Posterior probabilities of shifts in Thraupidae trait evolution identified by bayou

**Table 4** Parameter values of bayou output from the Thraupidae tree.

**Table 5** Comparisons of OUwie model fit for Thraupidae morphological traits

**Table 6** Beak morphology and diet correlations from GEE outputs

**Figure 1** (Top) Speciation rate through time plot of Sporophilinae (green) and Coerebinae (red) against that of Thraupidae (blue). (Bottom) Extinction rate through time plot of Sporophilinae (green) and Coerebinae (red) against that of Thraupidae (blue). 95% confidence intervals for speciation rates are given as shaded coloured areas. The dashed line indicates the origin of Darwin’s finches in the phylogeny.


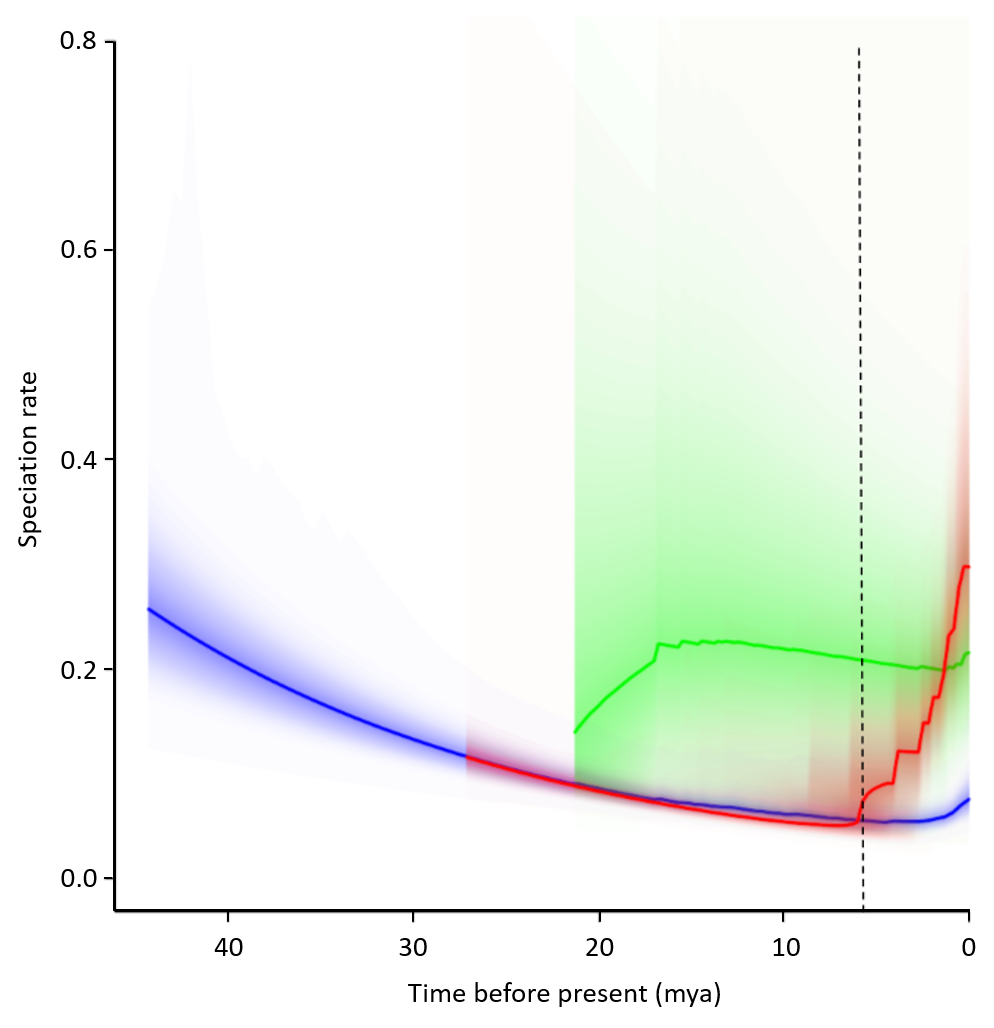

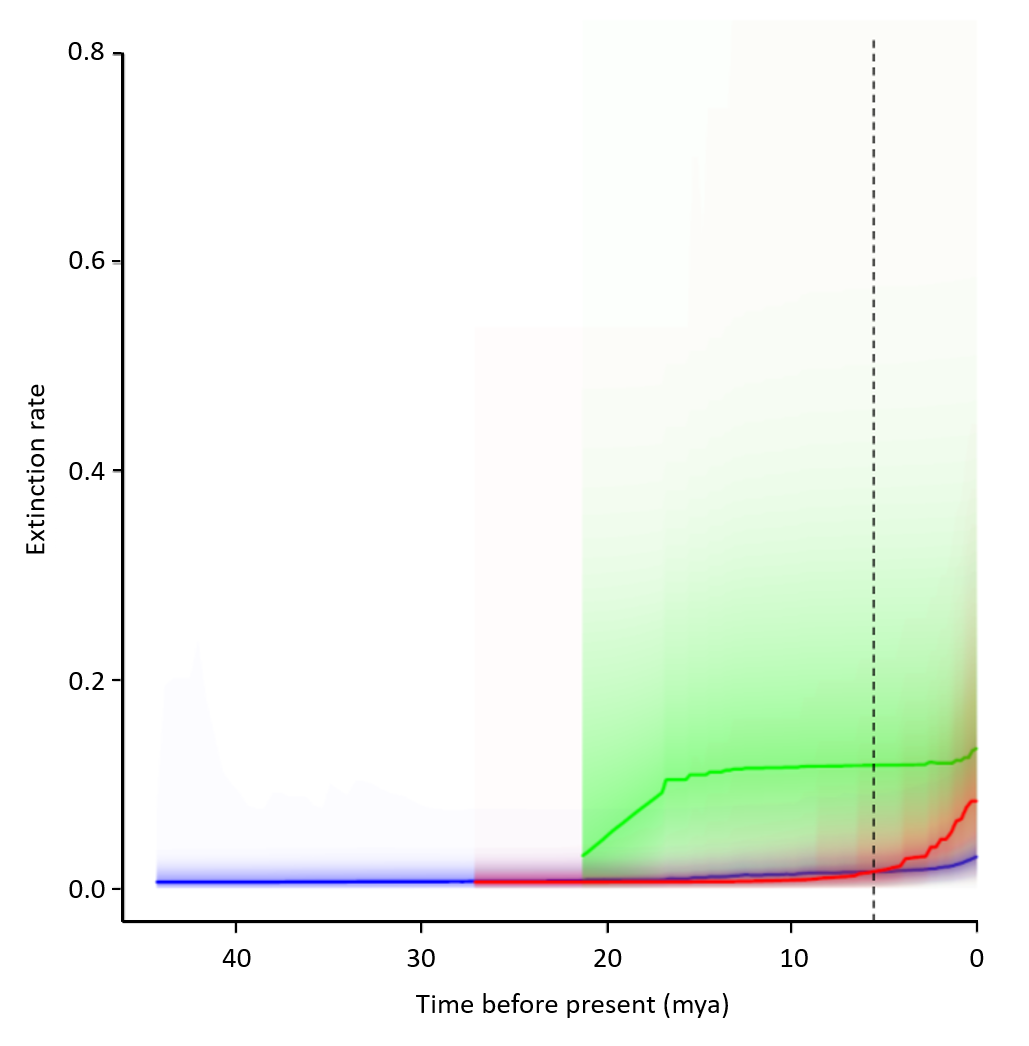


**Figure 2** Distinct shift configurations and their frequencies within the Thraupidae phylogeny. The frequencies given above each credible set indicate the most frequently sampled credible set of shift configurations from the BAMM analysis.


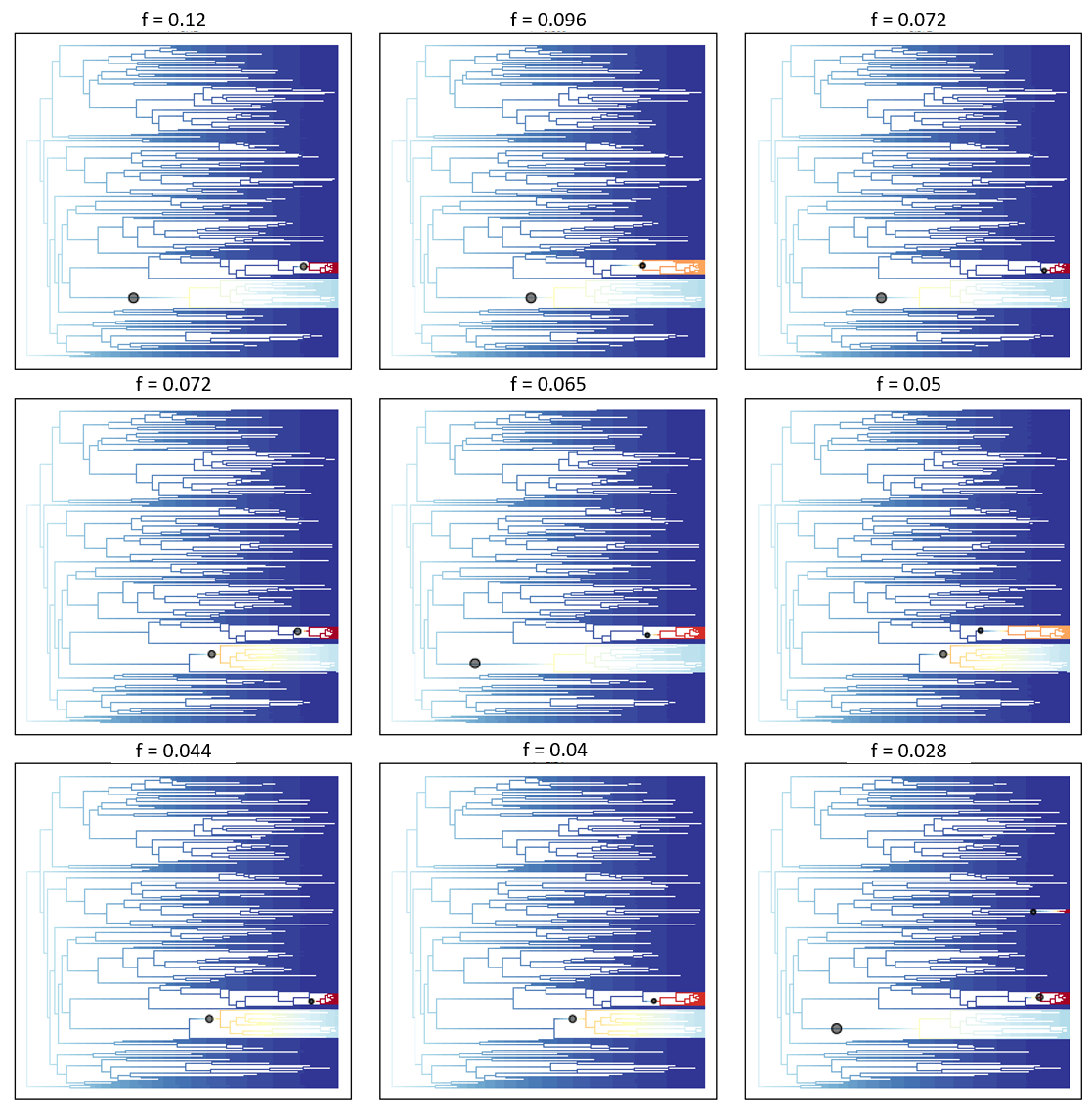


**Figure 3** Macroevolutionary cohort matrix displaying pairwise probabilities of shared common macroevolutionary rate regime. Species pairs sharing common rate dynamics show as red cells in the matrix and those with dissimilar or decoupled rates have blue matrix cells. Coerebinae and Sporophilinae do not share a common rate dynamic due to the existence of a rate shift on their respective ancestral branches (rate shift not shown here but see figure 1 in the article). Thraupidae phylogeny is shown above and to the left of the matrix.


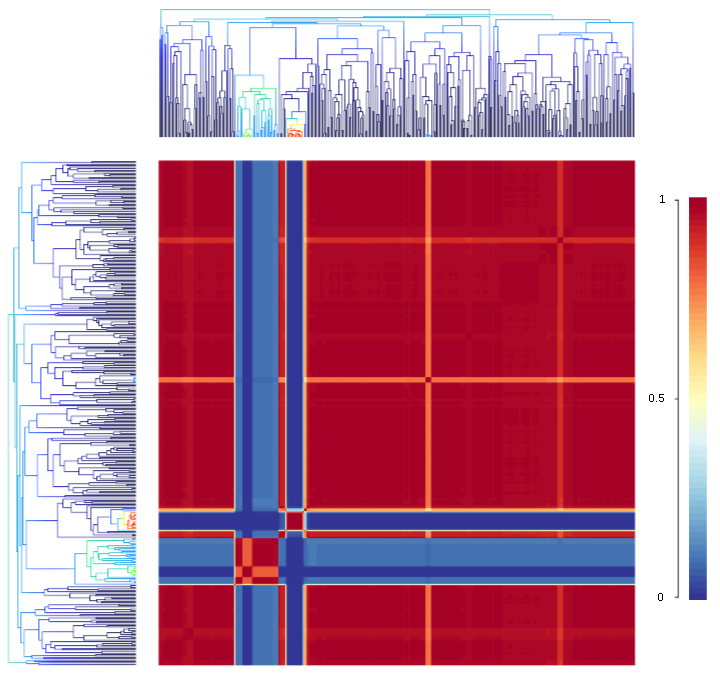


**Figure 4** Trait simmap of Coerebinae, Sporophilinae and outgroups with branches coloured according to trait regimes for the first beak PCA values. Size of red-filled circles illustrates the probability of the shift occurring on the branch. Posterior probability cut-off for all regimes is set to 0.5. Subfamilies are given above the phylogeny tips with notable subclades highlighted in red with Caribbean bullfinches (*), Darwin’s finches (ψ) and *Oryzoborus* (β). Histogram of raw trait values are shown above the clade names.

**
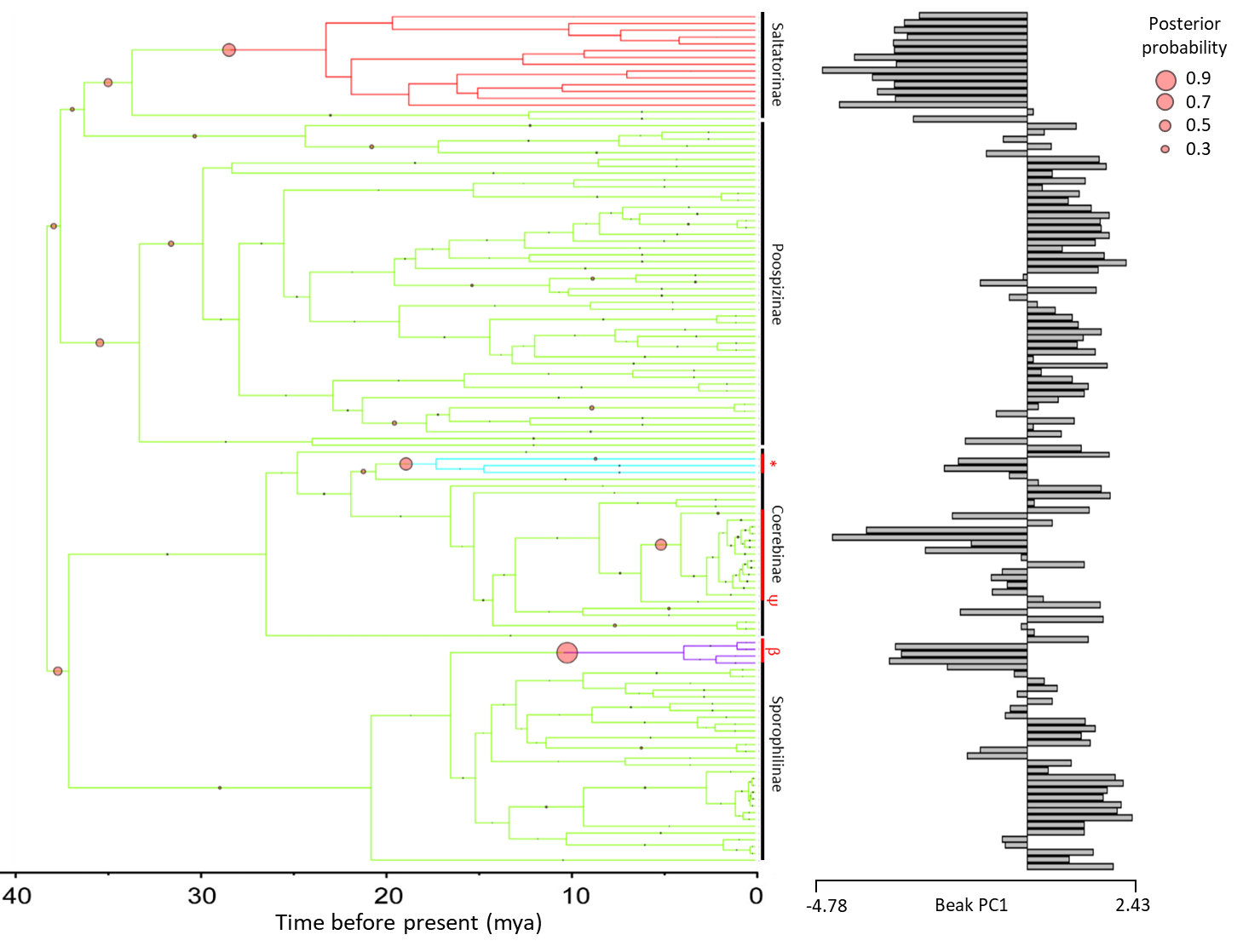
**

**Figure 5** Trait simmap of Coerebinae, Sporophilinae and outgroups with branches coloured according to trait regimes for body mass values. All subclade symbols, trait regimes and posterior probability are as described in Fig. S7.

**
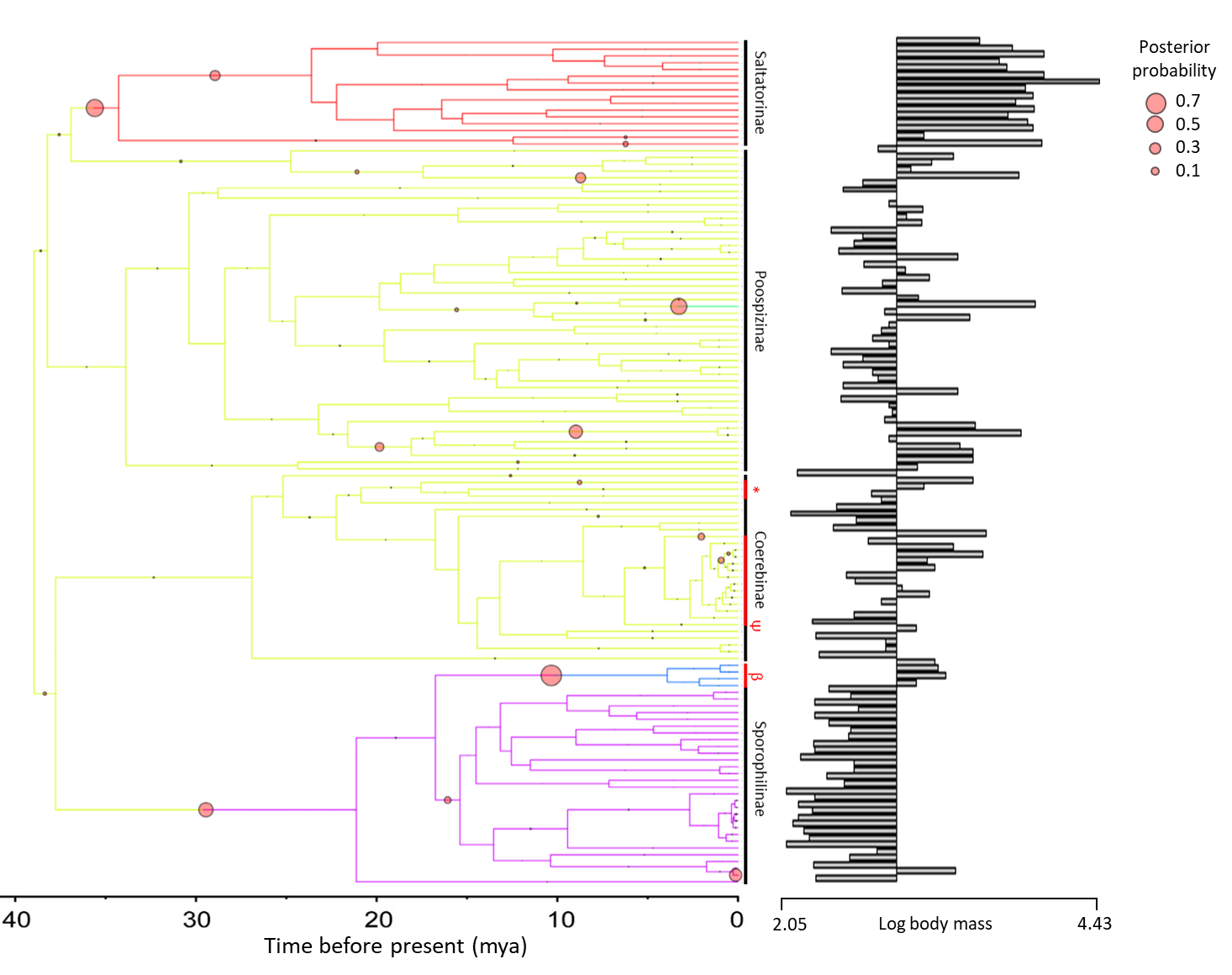
**

**Figure 6** Trait simmap of Coerebinae, Sporophilinae and outgroups with branches coloured according to trait regimes for culmen length values. All subclade symbols, trait regimes and posterior probability are as described in Fig. S7.

**
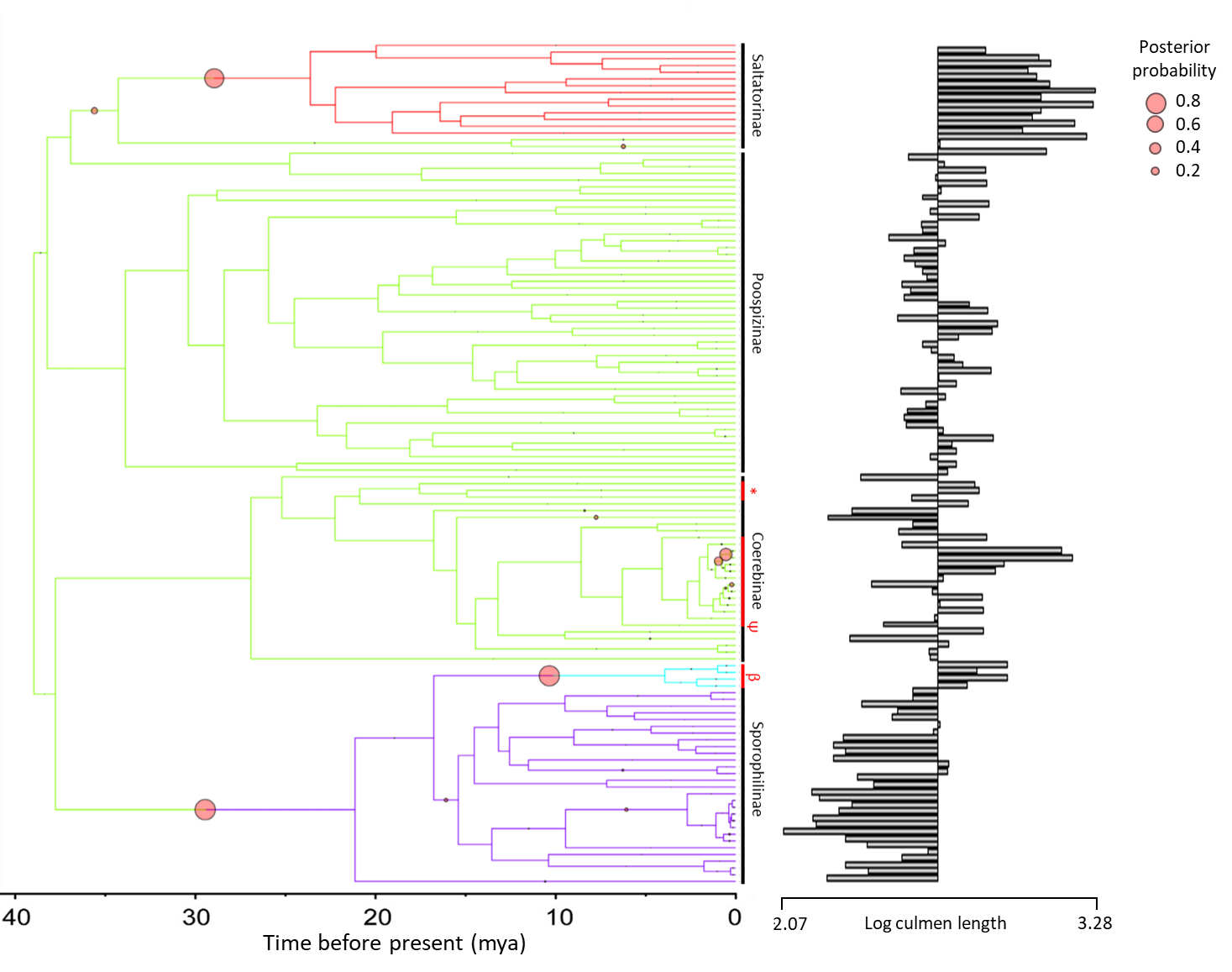
**

**Figure 7** Trait simmap of Coerebinae, Sporophilinae and outgroups with branches coloured according to trait regimes for beak tip to nares length values. All subclade symbols, trait regimes and posterior probability are as described in Fig. S7.

**
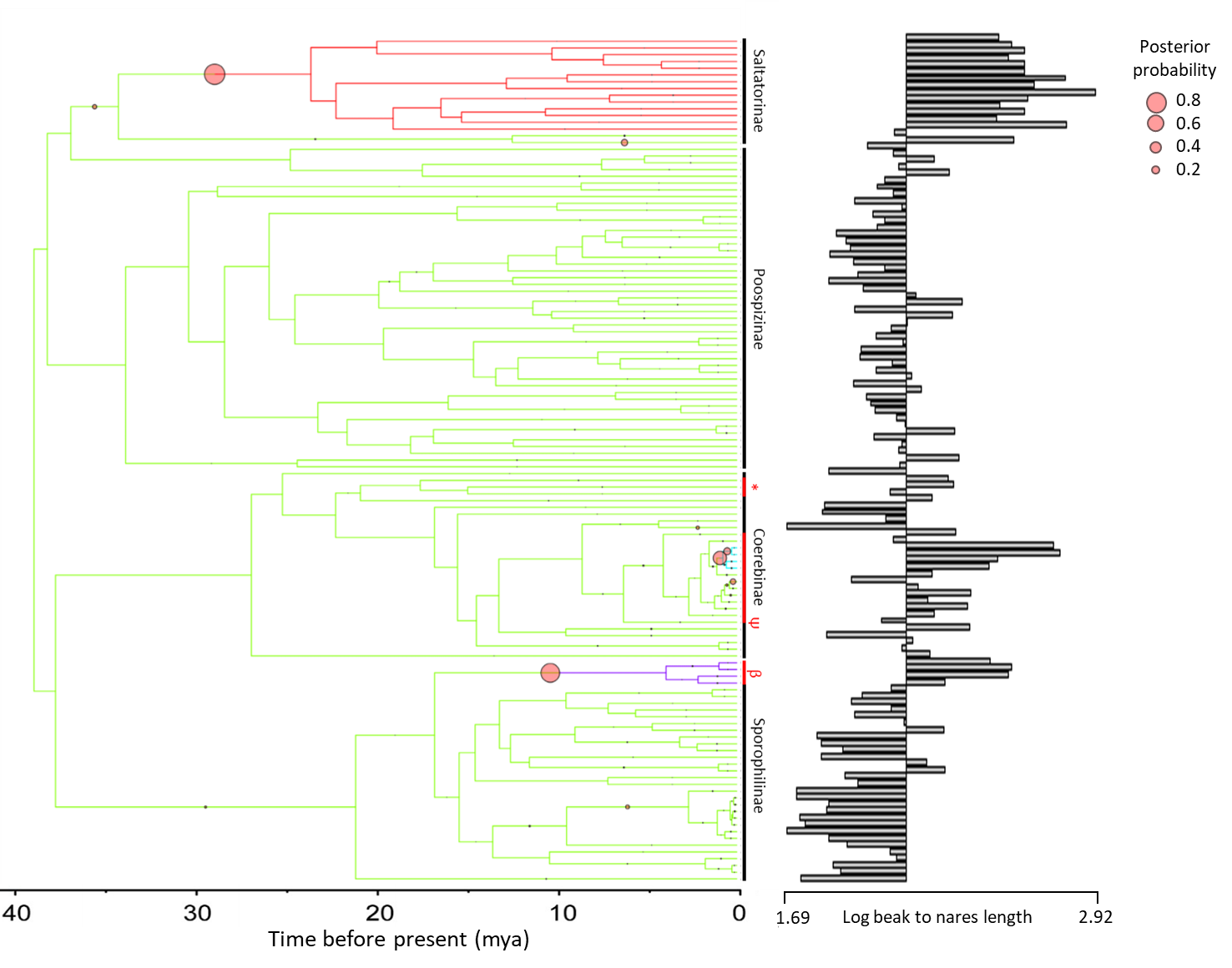
**

**Figure 8** Trait simmap of Coerebinae, Sporophilinae and outgroups with branches coloured according to trait regimes for beak width values. All subclade symbols, trait regimes and posterior probability are as described in Fig. S7.

**
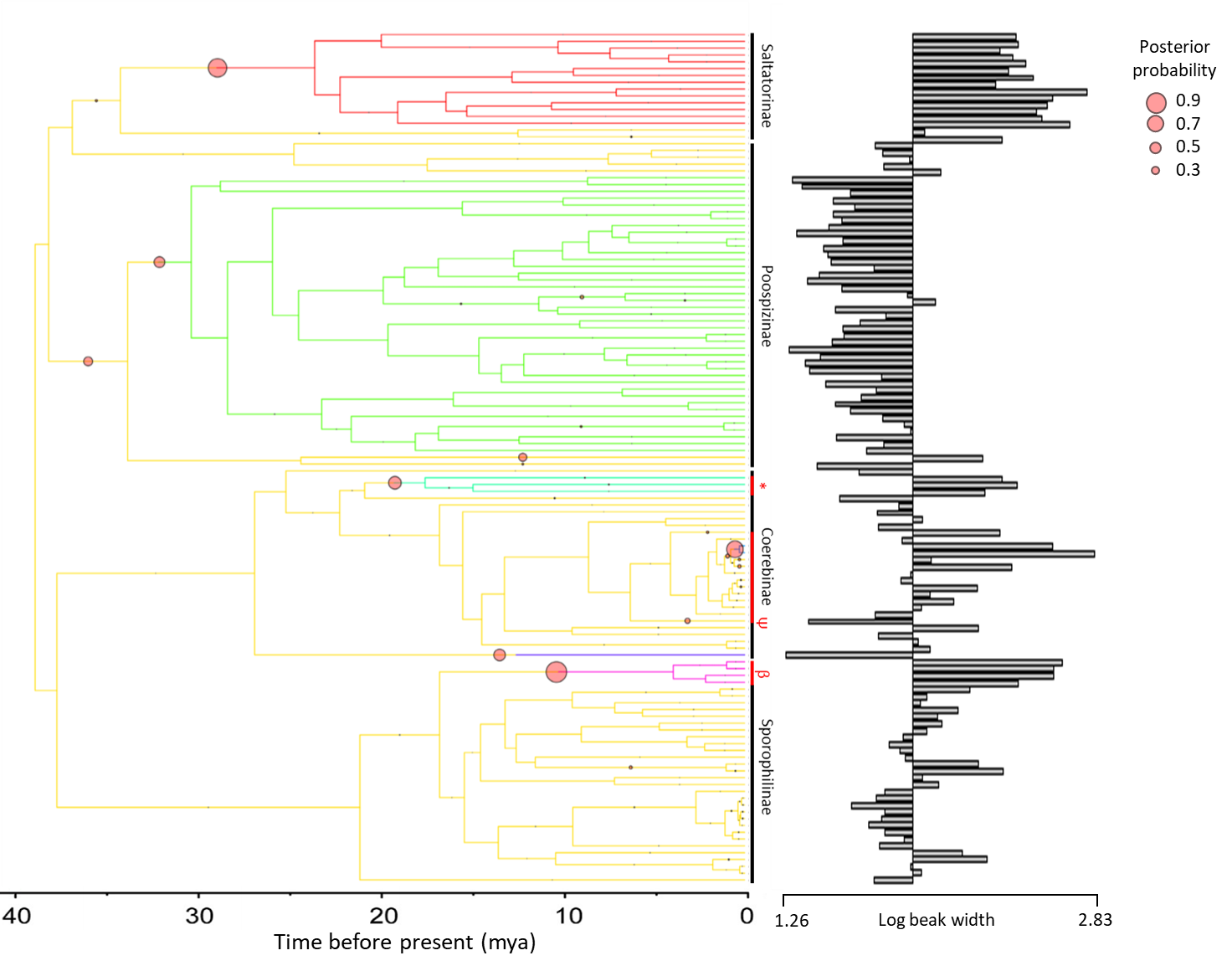
**

**Figure 9** Trait simmap of Coerebinae, Sporophilinae and outgroups with branches coloured according to trait regimes for beak depth values. All subclade symbols, trait regimes and posterior probability are as described in Fig. S7.

**
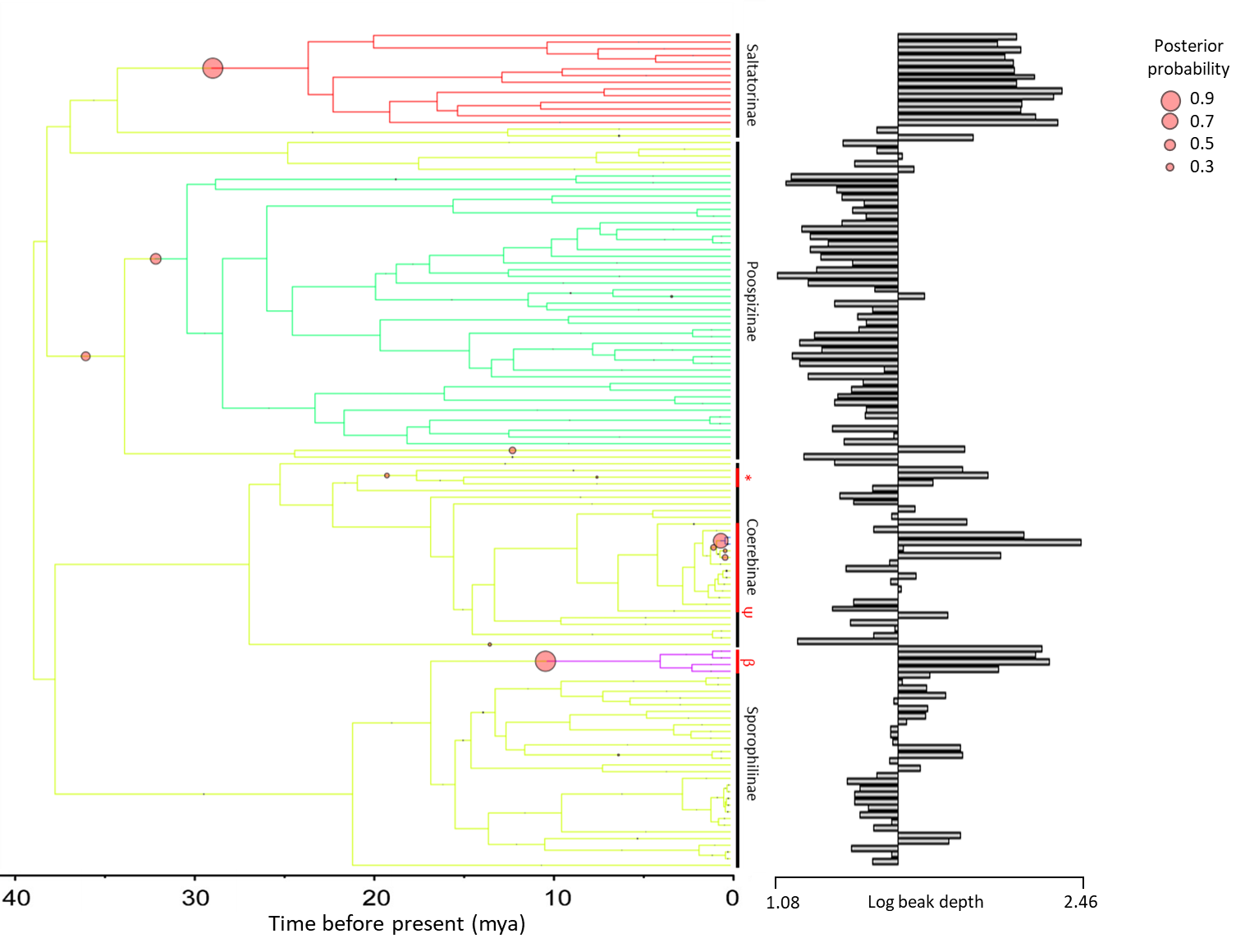
**

**Figure 10** Trait simmap of Coerebinae, Sporophilinae and outgroups with branches coloured according to trait regimes for tarsus length values. All subclade symbols, trait regimes and posterior probability are as described in Fig. S7.

**
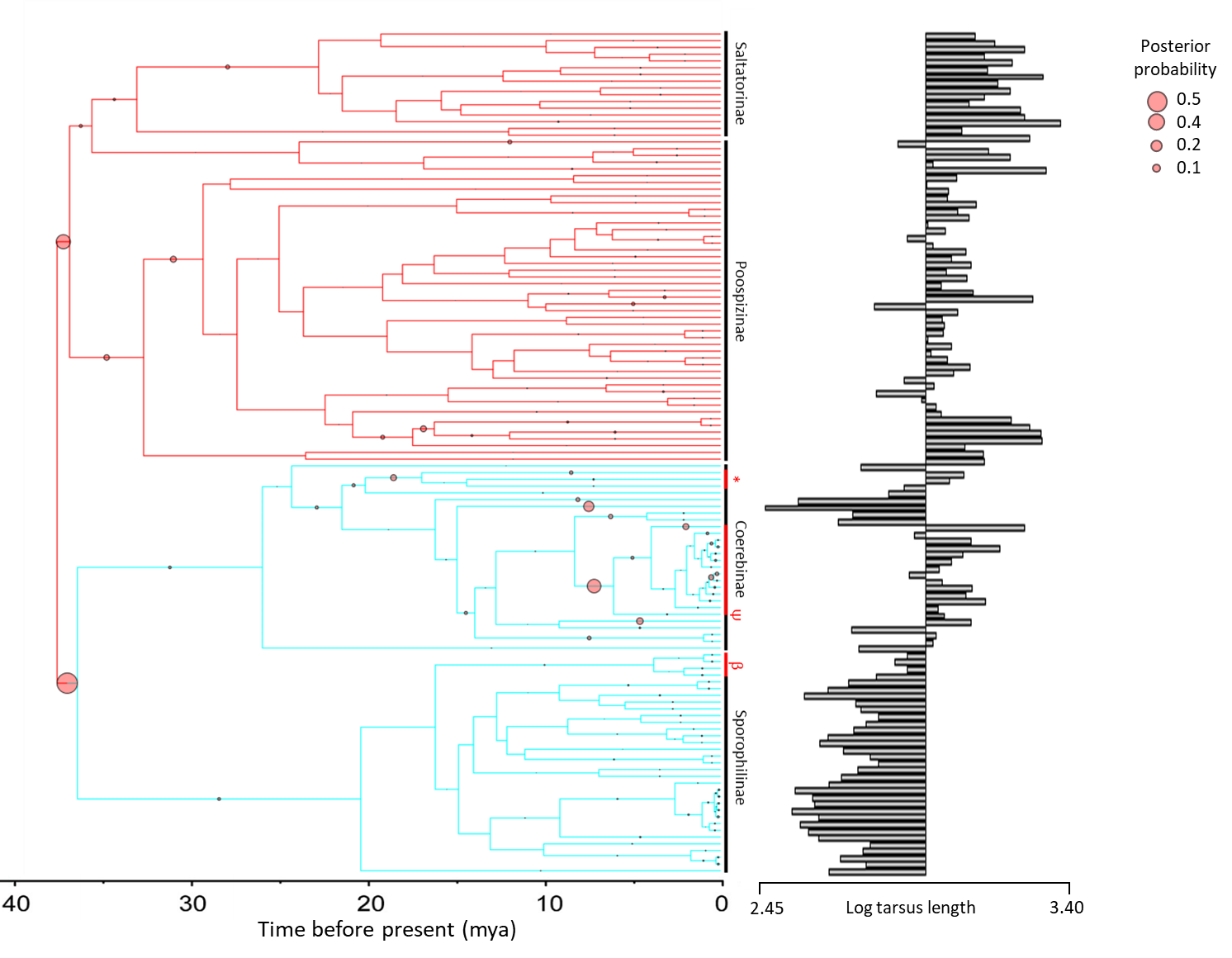
**

**Figure 11** Trait simmap of Coerebinae, Sporophilinae and outgroups with branches coloured according to trait regimes for hand-wing index values. All subclade symbols, trait regimes and posterior probability are as described in Fig. S7.

**
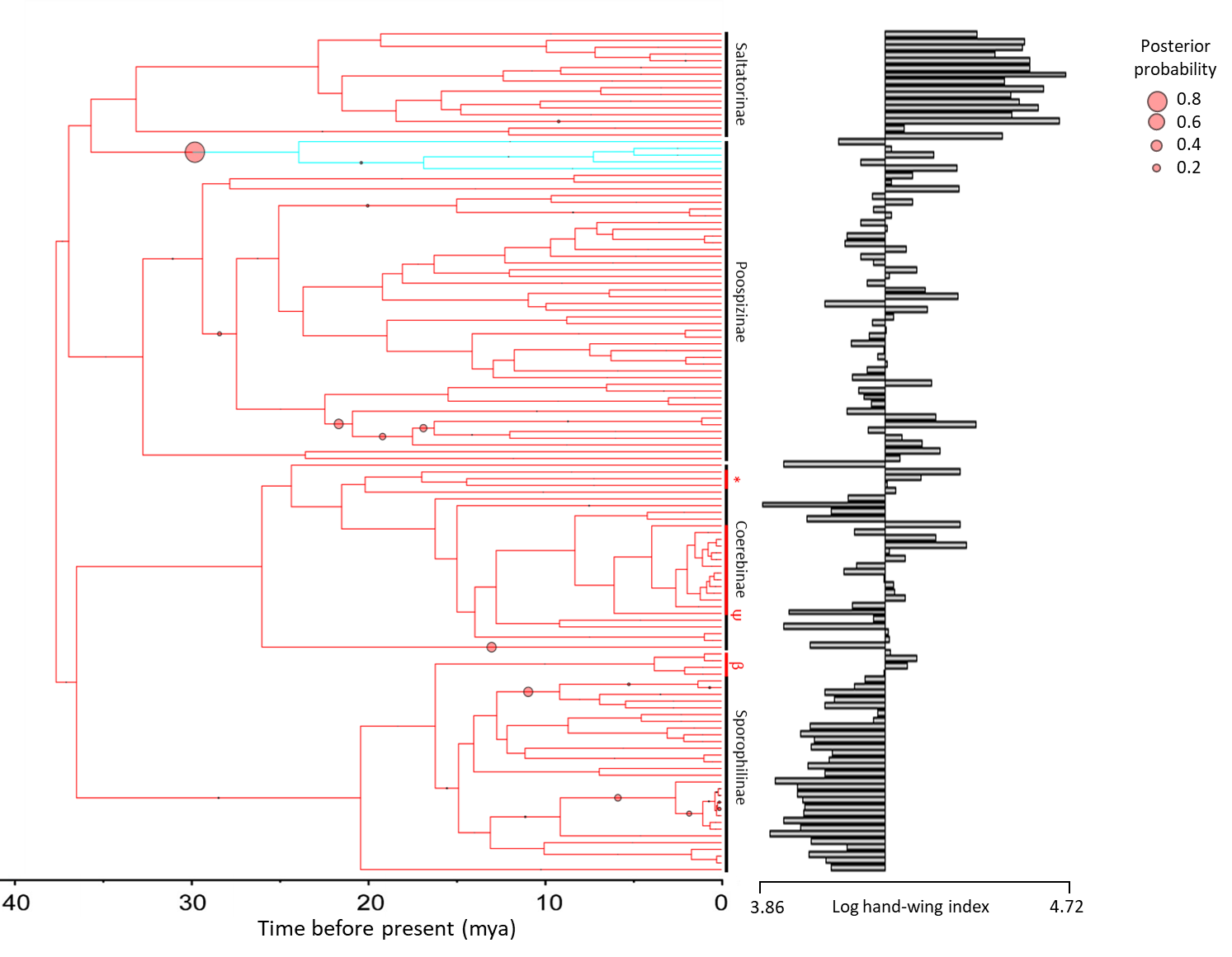
**

**Figure 12** Trait simmap of Coerebinae, Sporophilinae and outgroups with branches coloured according to trait regimes for wing chord length values. All subclade symbols, trait regimes and posterior probability are as described in Fig. S7.

**
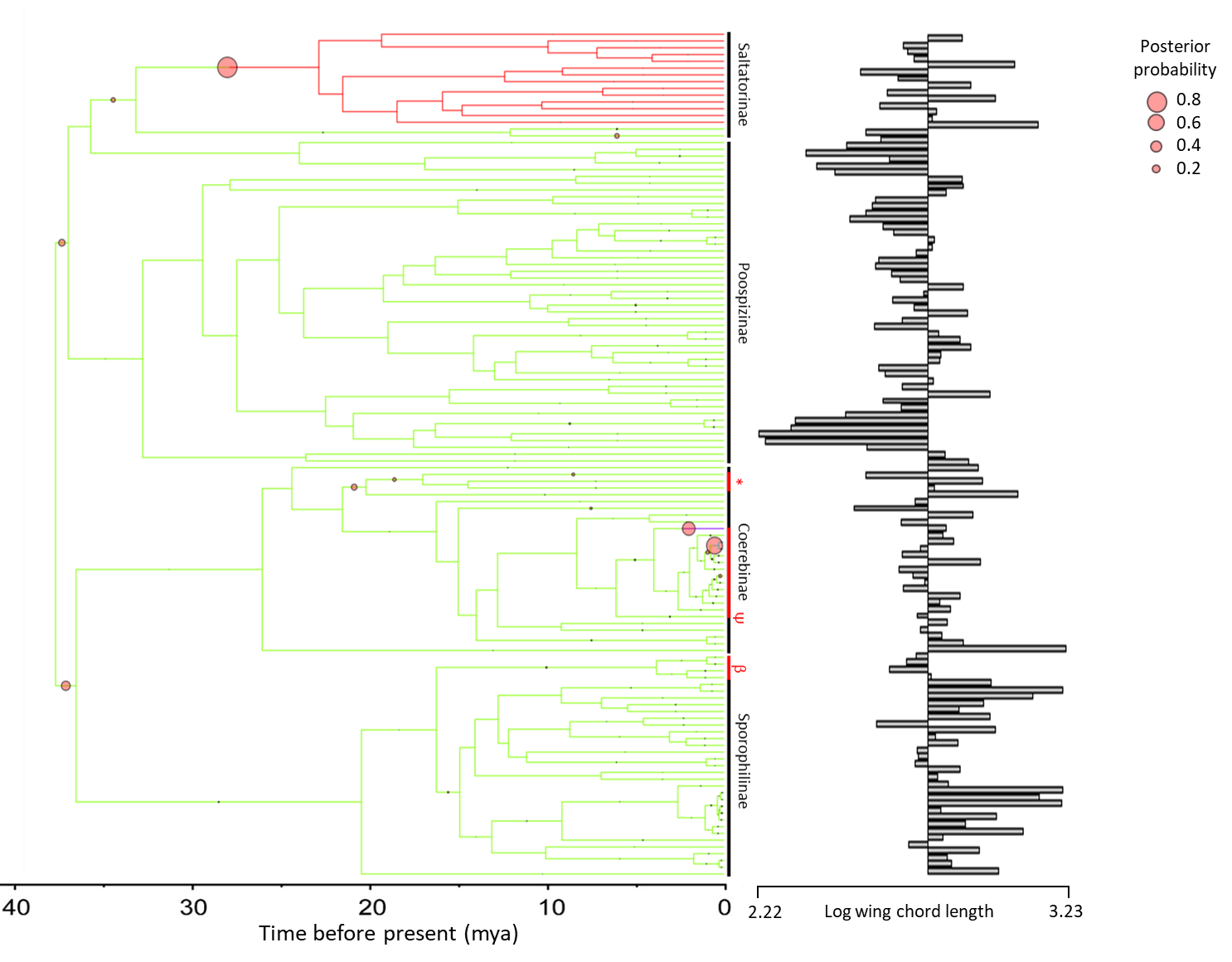
**

**Figure 13** Trait simmap of Coerebinae, Sporophilinae and outgroups with branches coloured according to trait regimes for tail length values. All subclade symbols, trait regimes and posterior probability are as described in Fig. S7.

**
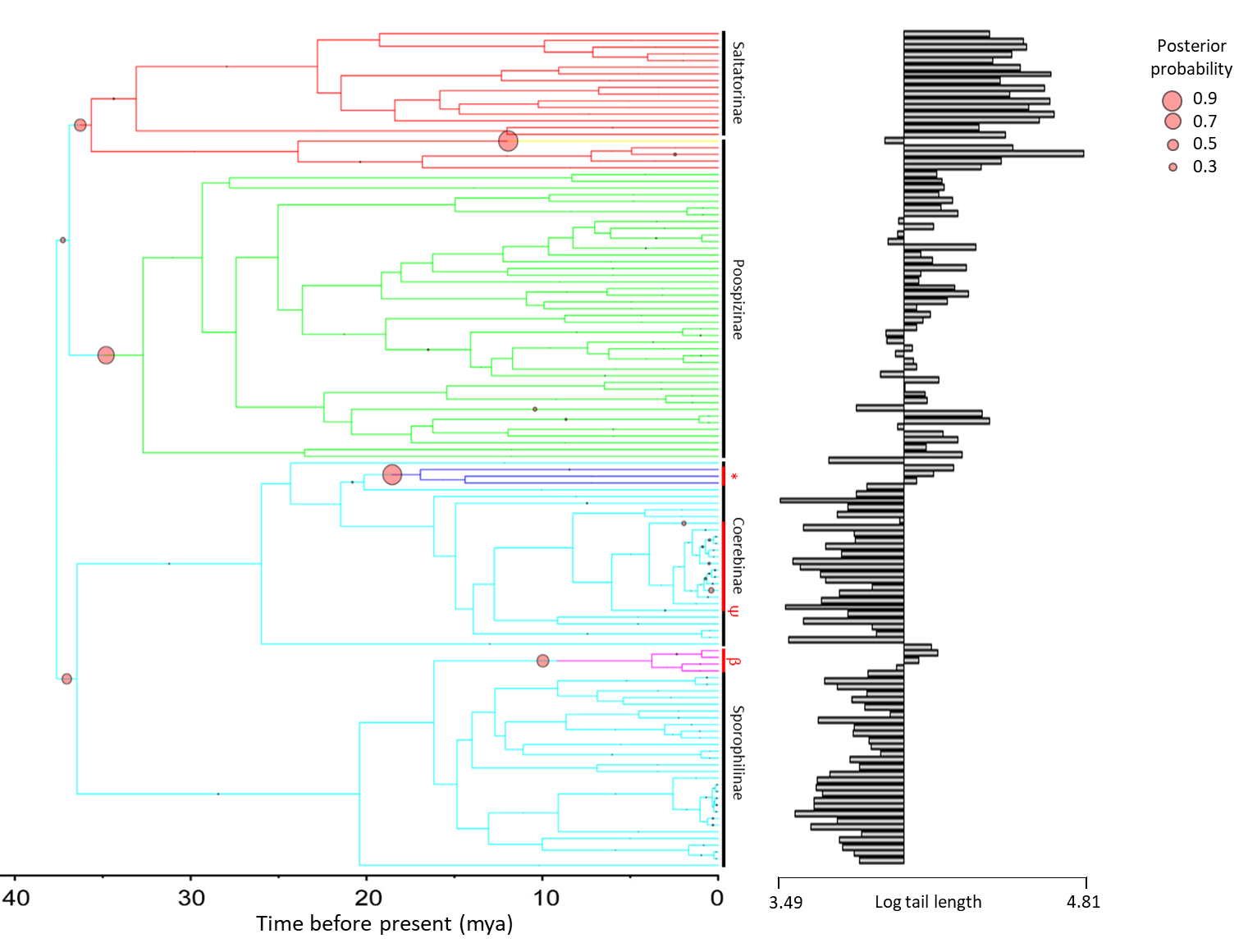
**

**Figure 14** Simmap of the Thraupidae phylogeny with branches coloured according to trait regimes. Size of red-filled circles illuatrates the probability of the shift occurring on the branch. The posterior probability cut-off for all regimes is set to 0.5, with the exception of wing-chord length and tail length which were set to 0.2 and 0.4, respectively. Histogram of raw trait values are shown above the phylogeny. Clades are given as abbreviations; Thraupinae (Th), Porphyrospizinae (Pr), Nemosiinae (Ne), Diglossinae (Di), Saltatorinae (Sa), Emberizoidinae (Em), Poospizinae (Po), Coerebinae (Co), Sporophilinae (Sp), Dacninae (Da), Hemithraupinae (He) and Tachyphoninae (Ta). Clades Charitospizinae, Catamblyrhynchinae and Orchesticinae are included but not labelled due to very low number of taxa.


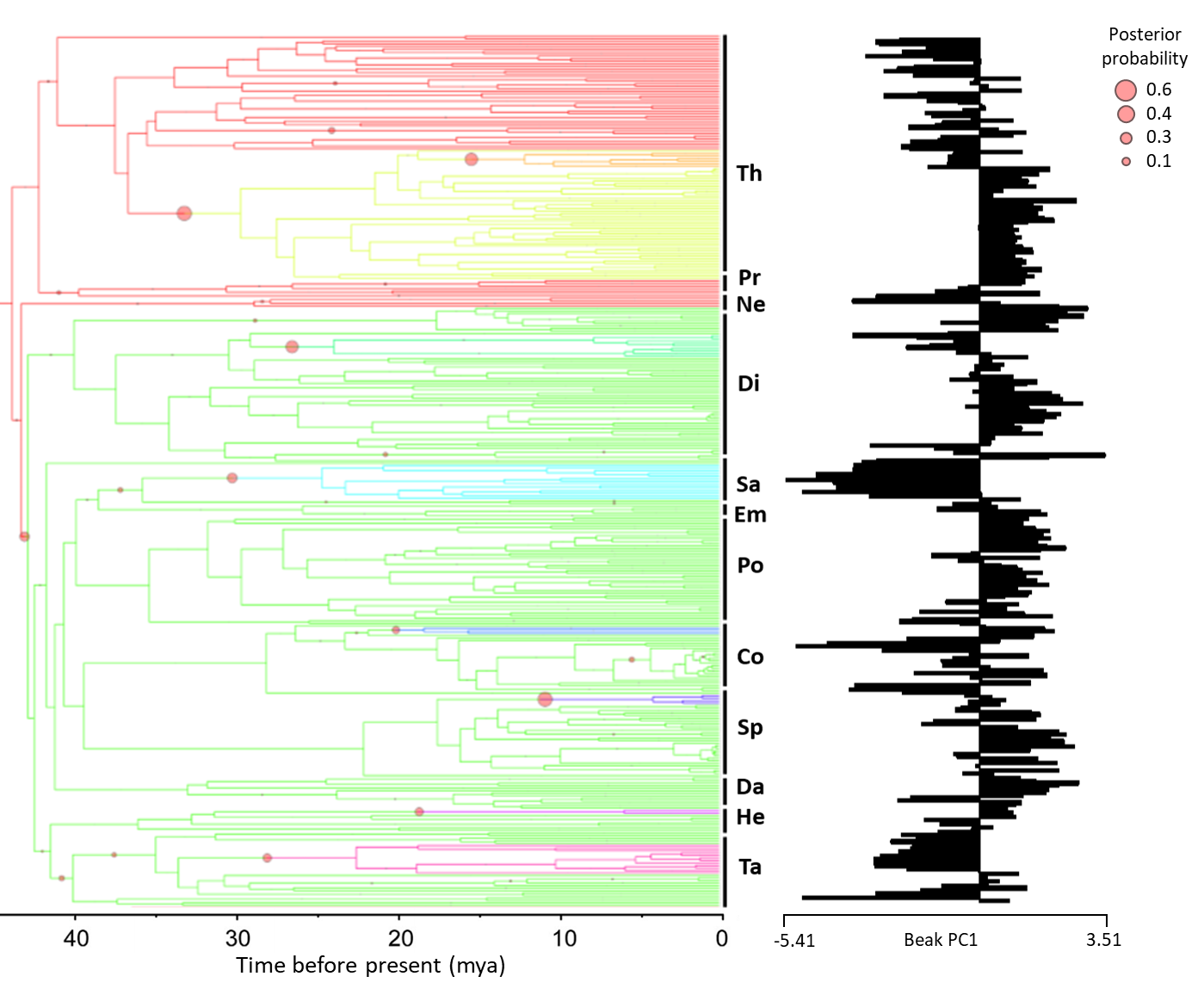


**Figure 15** Simmap of the Thraupidae phylogeny with branches coloured according to trait regimes for body mass values. All clade labels, trait regimes and posterior probability are as described in Fig. S17.


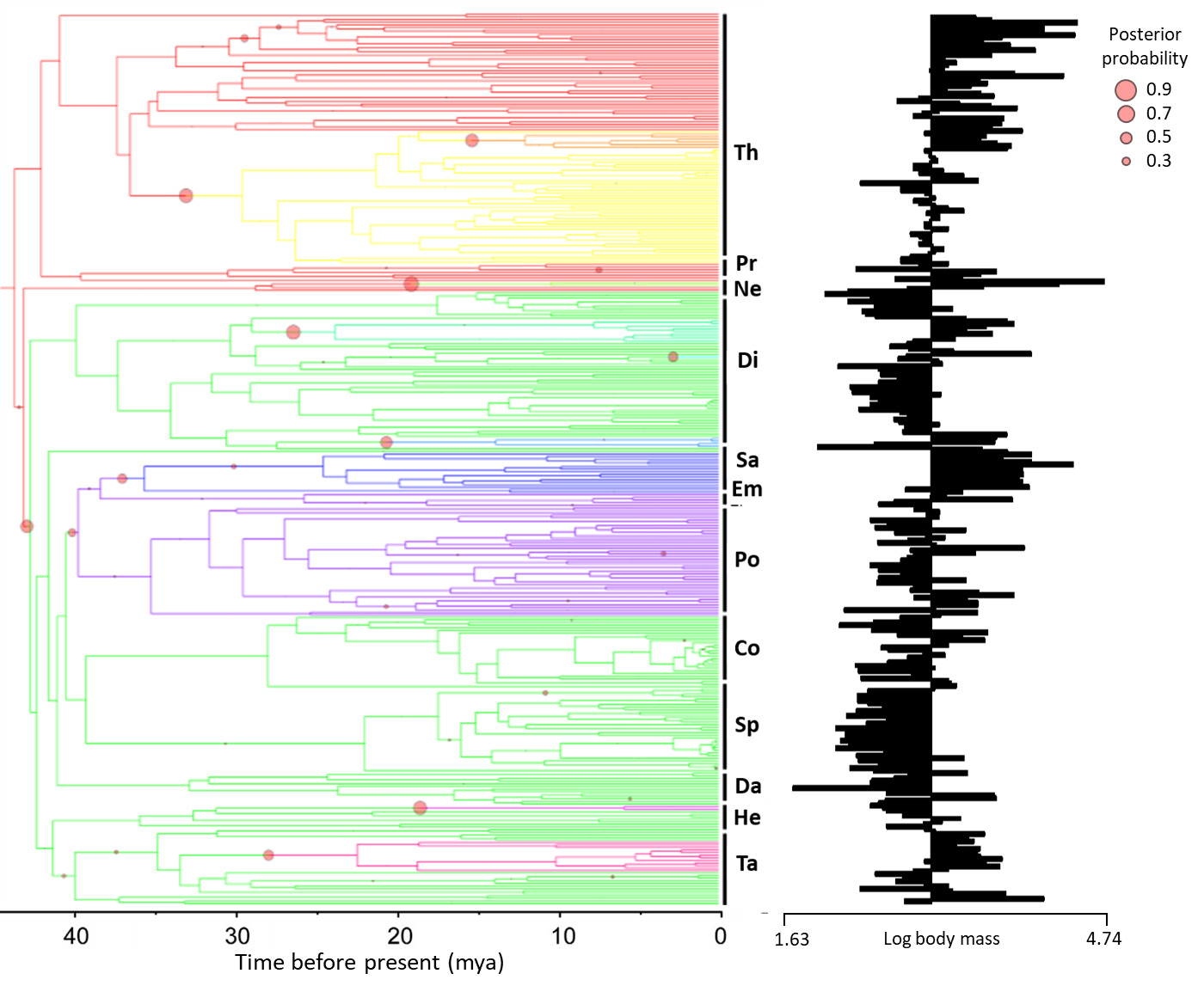


**Figure 16** Simmap of the Thraupidae phylogeny with branches coloured according to trait regimes for culmen length values. All clade labels, trait regimes and posterior probability are as described in Fig. S17.


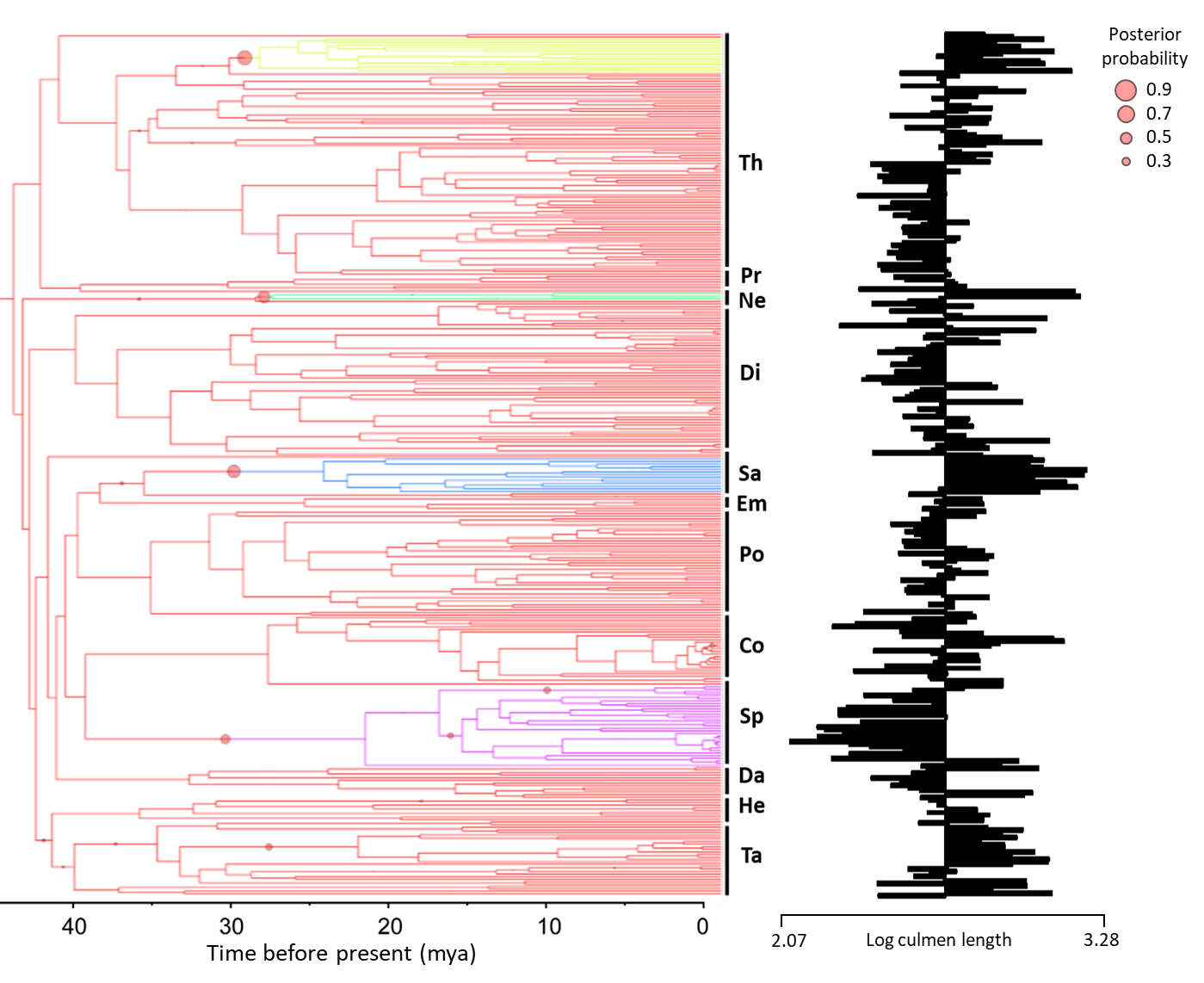


**Figure 17** Simmap of the Thraupidae phylogeny with branches coloured according to trait regimes for beak tip to nares length values. All clade labels, trait regimes and posterior probability are as described in Fig. S17.


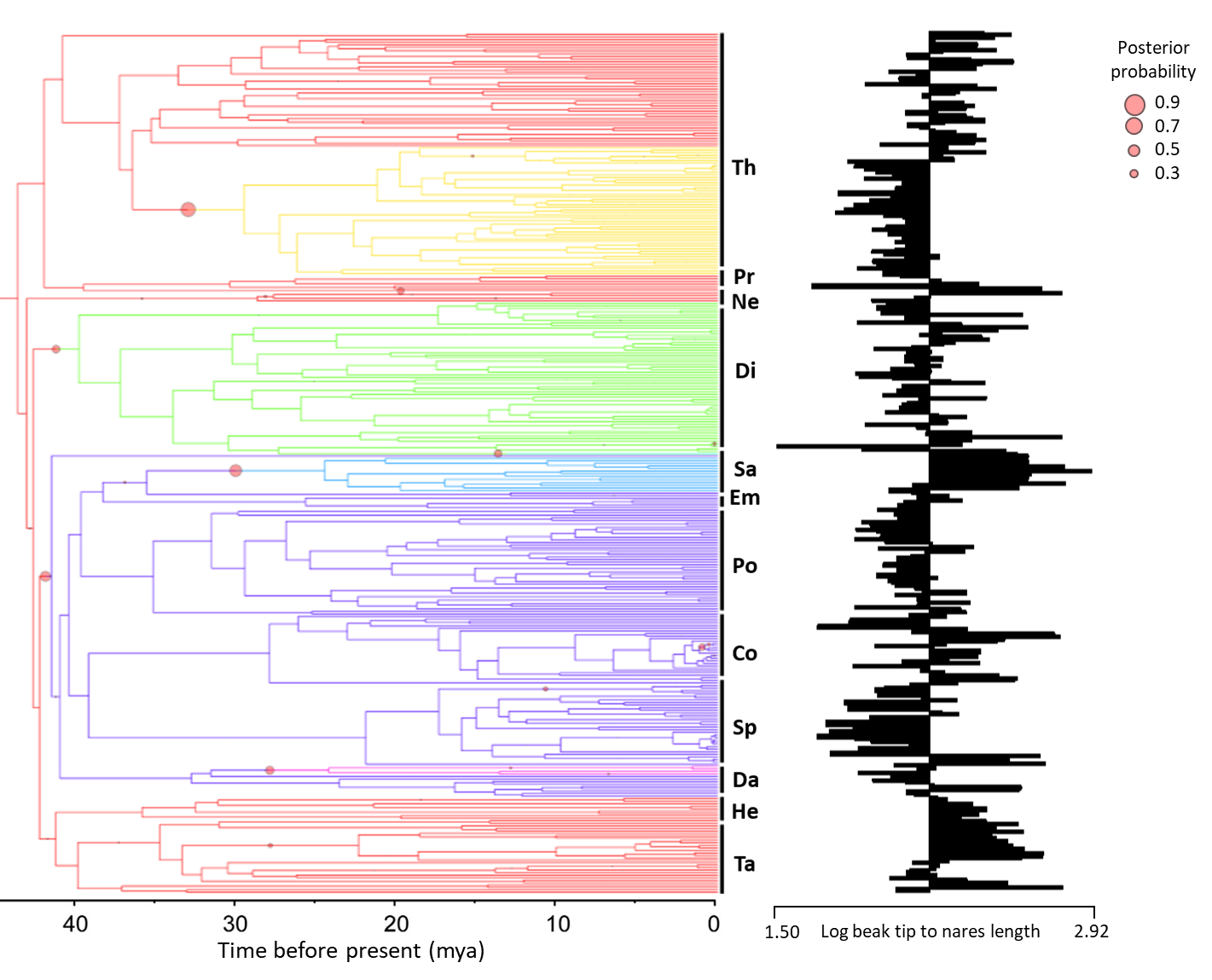


**Figure 18** Simmap of the Thraupidae phylogeny with branches coloured according to trait regimes for beak width values. All clade labels, trait regimes and posterior probability are as described in Fig. S17.


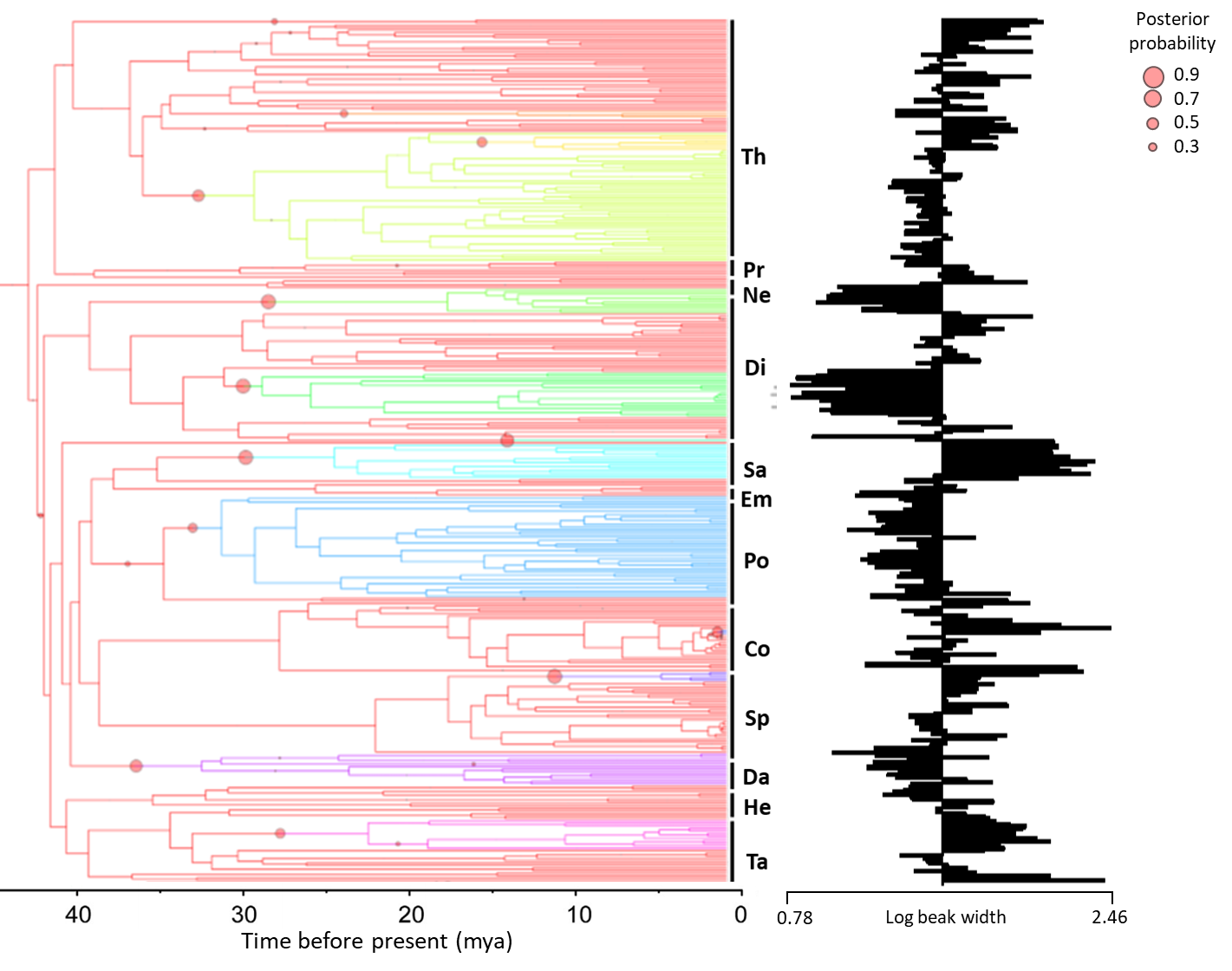


**Figure 19** Simmap of the Thraupidae phylogeny with branches coloured according to trait regimes for beak depth values. All clade labels, trait regimes and posterior probability are as described in Fig. S17.


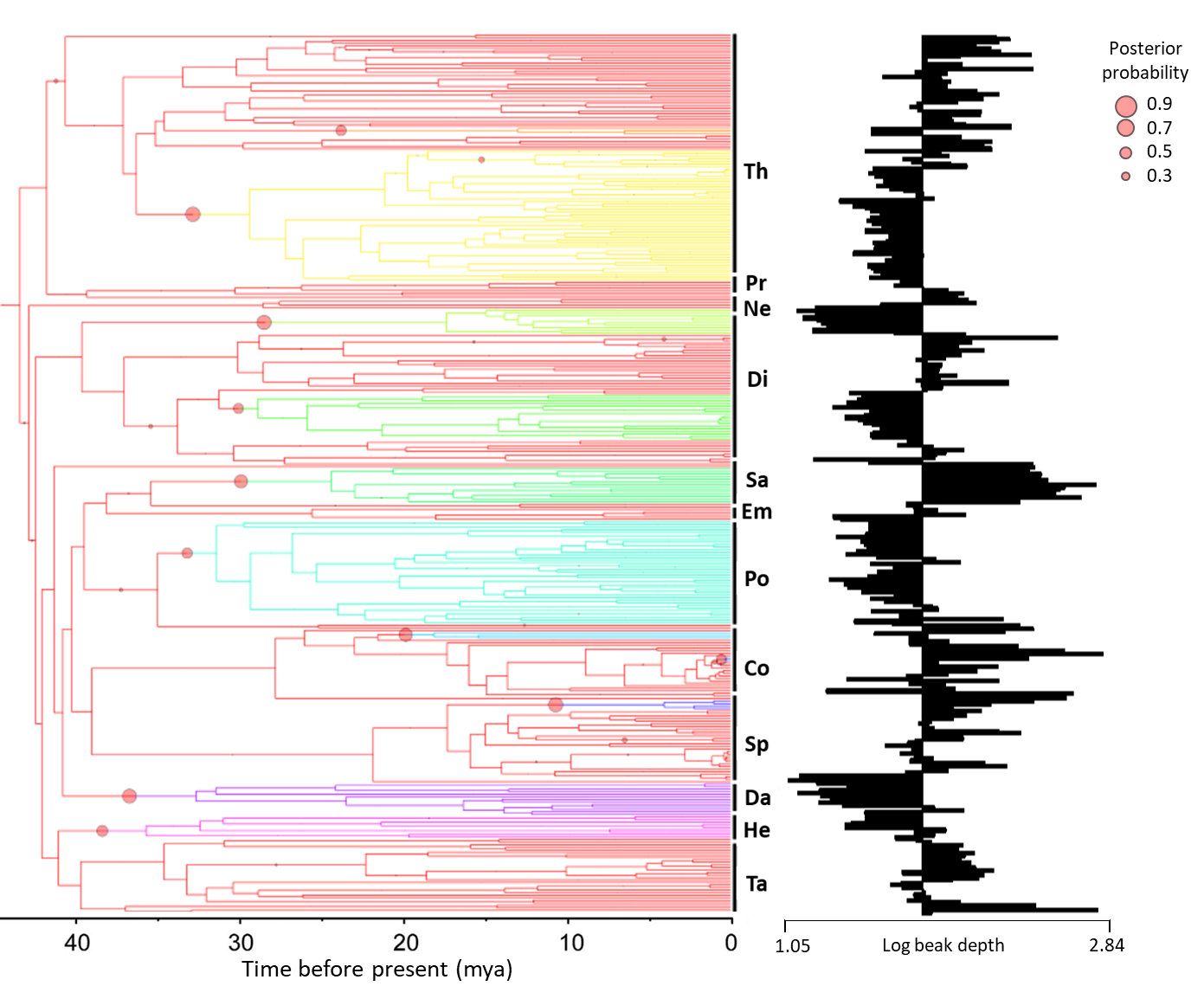


**Figure 20** Simmap of the Thraupidae phylogeny with branches coloured according to trait regimes for tarsus length values. All clade labels, trait regimes and posterior probability are as described in Fig. S17.


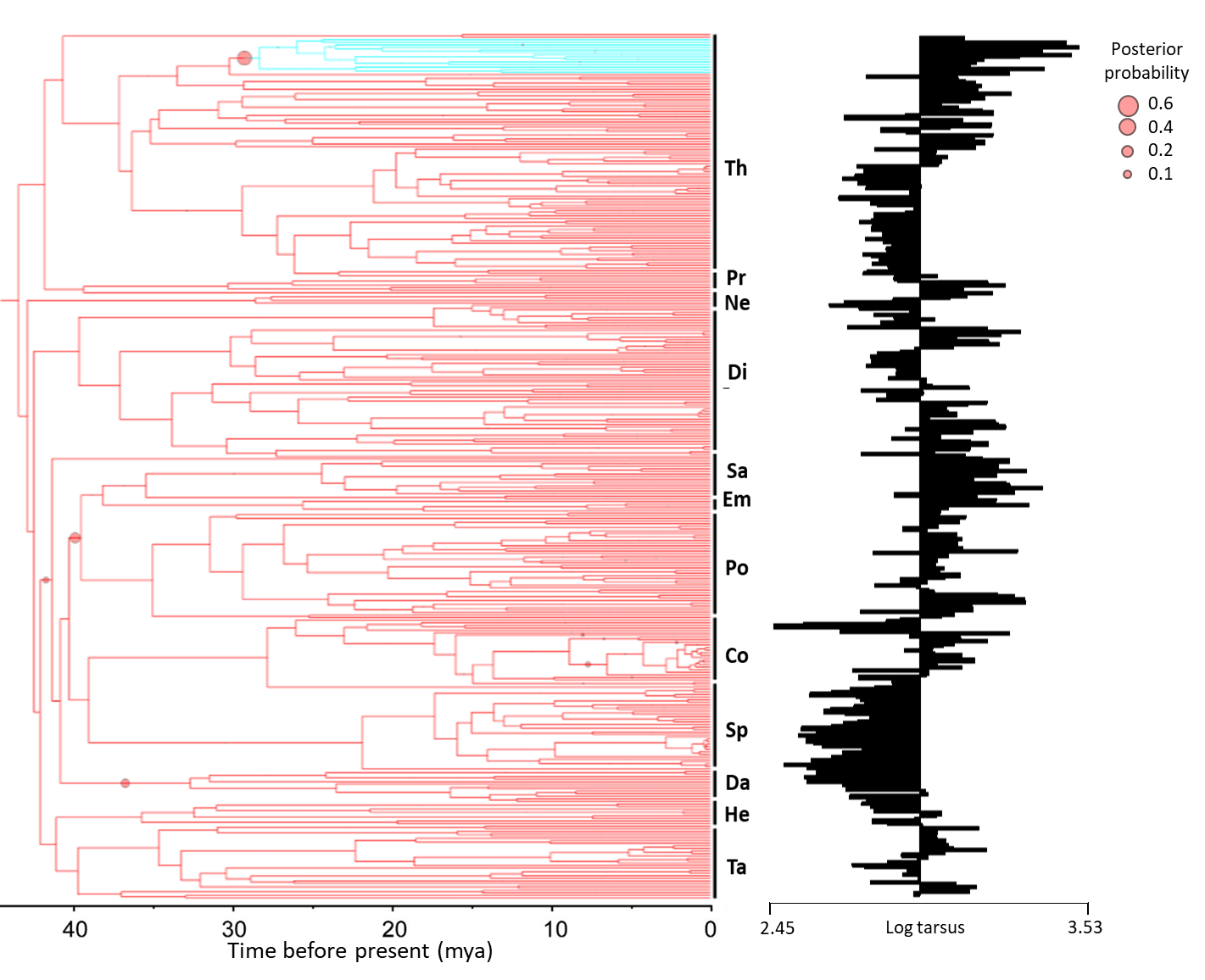


**Figure 21** Simmap of the Thraupidae phylogeny with branches coloured according to trait regimes for wing-chord length values. All clade labels, trait regimes and posterior probability are as described in Fig. S17.


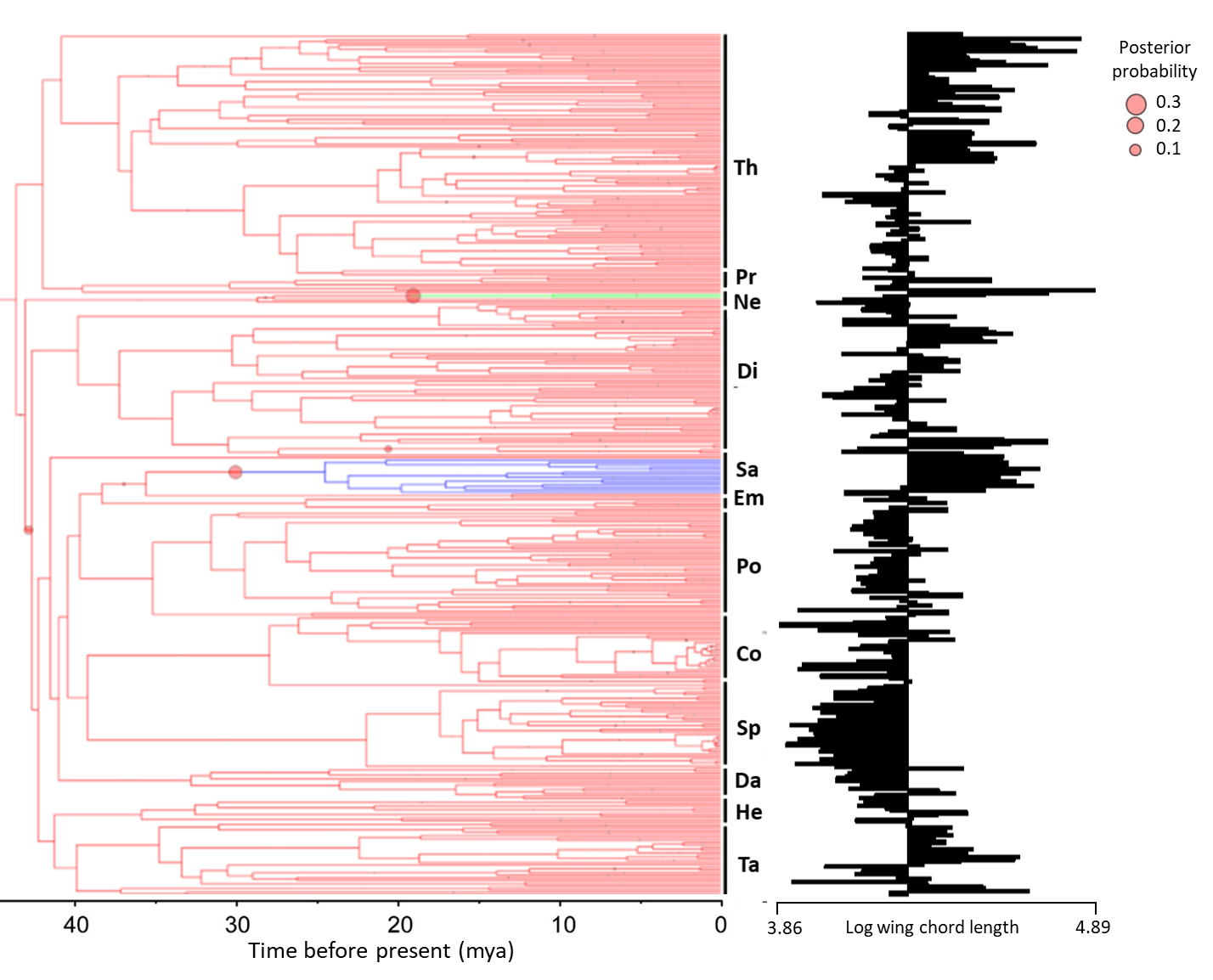


**Figure 22** Simmap of the Thraupidae phylogeny with branches coloured according to trait regimes for hand-wing index values. All clade labels, trait regimes and posterior probability are as described in Fig. S17.


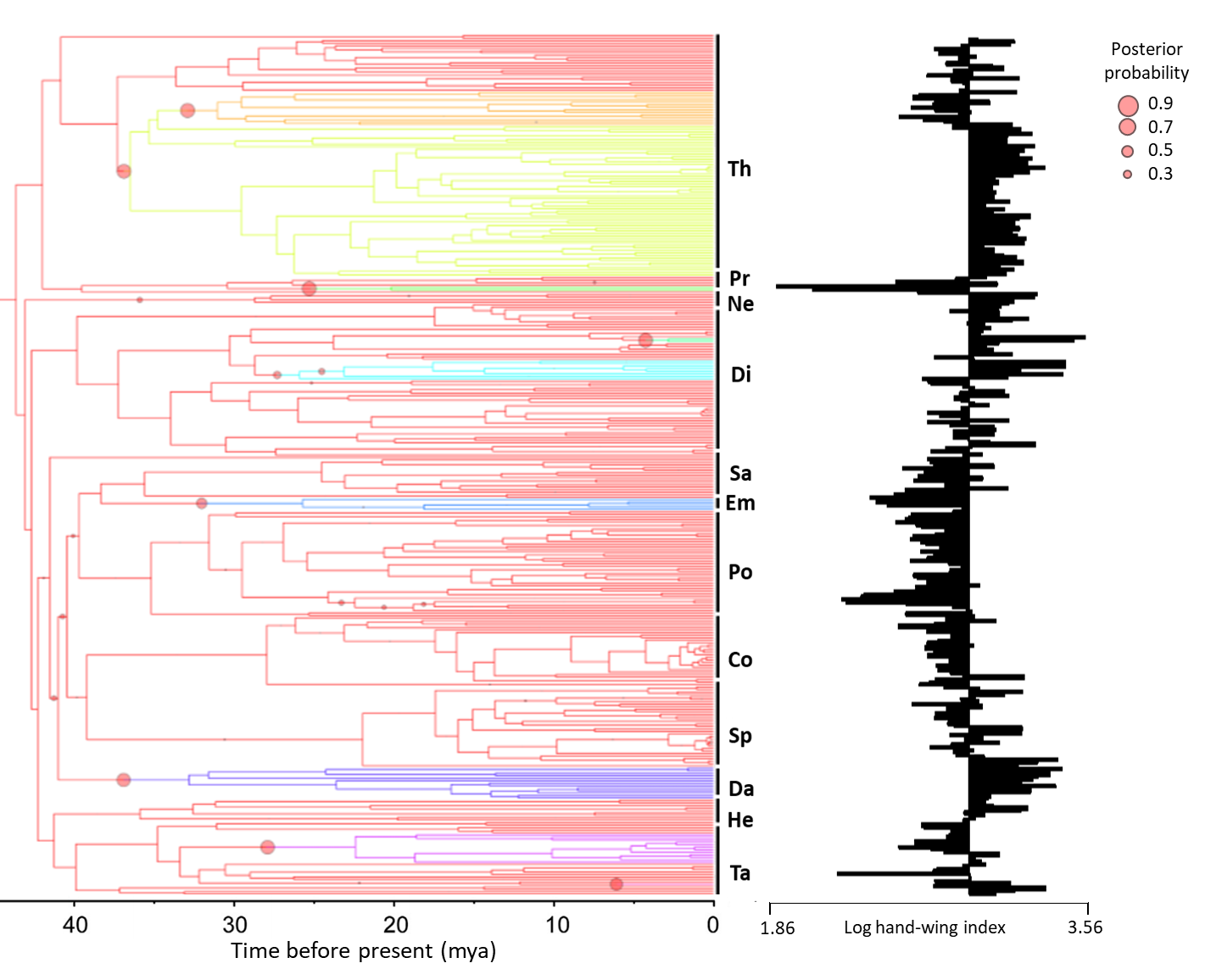


**Figure 23** Simmap of the Thraupidae phylogeny with branches coloured according to trait regimes for tail length values. All clade labels, trait regimes and posterior probability are as described in Fig. S17.


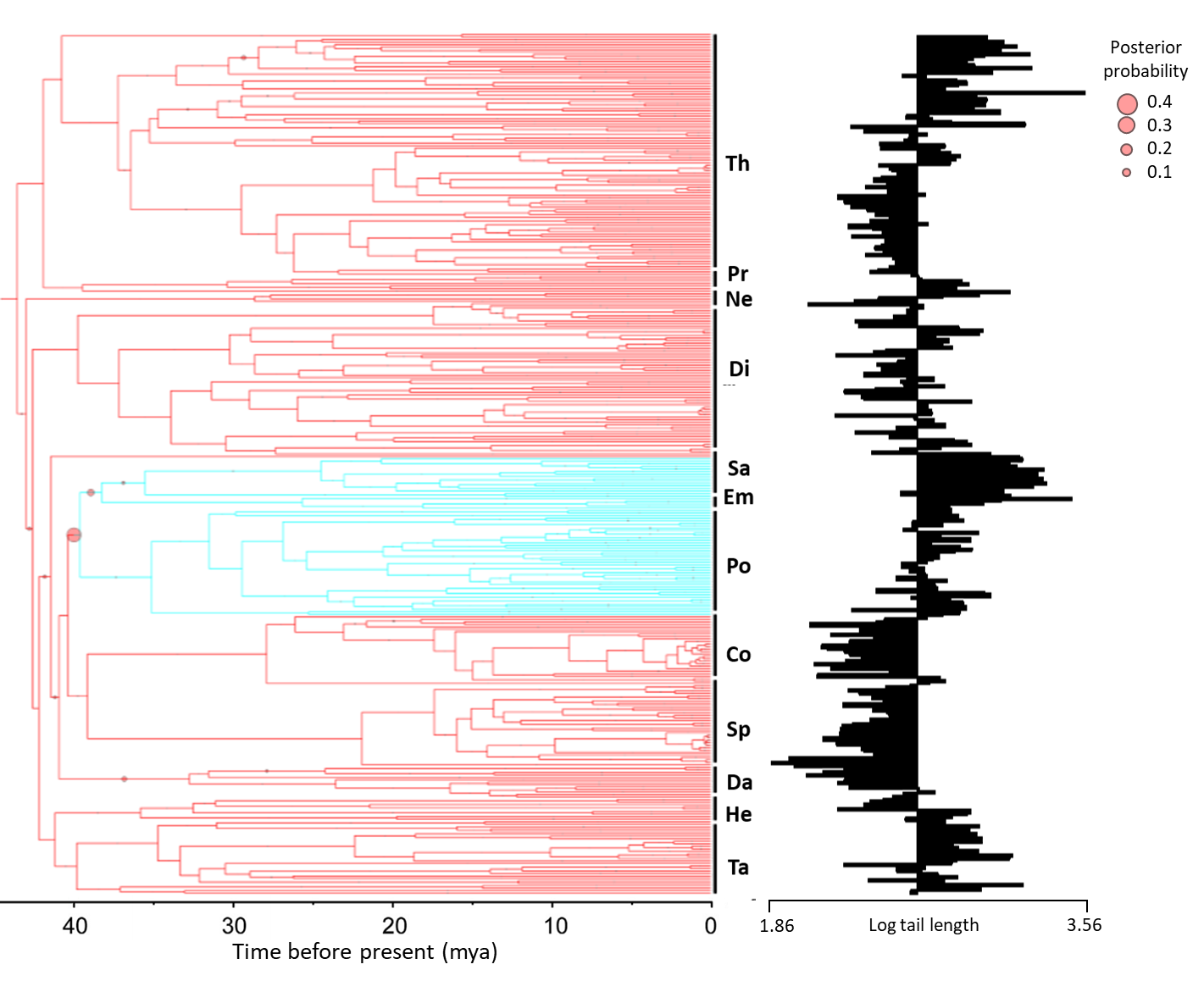


**Figure 24** PCoA plot of dietary composition values of Thraupidae, Coerebinae and Sporophilinae based on Euclidean distances. Points correspond to the major dietary component (i.e. the food-type consisting of 60% of species’ overall dietary makeup) with areas shaded according to taxonomic clade.


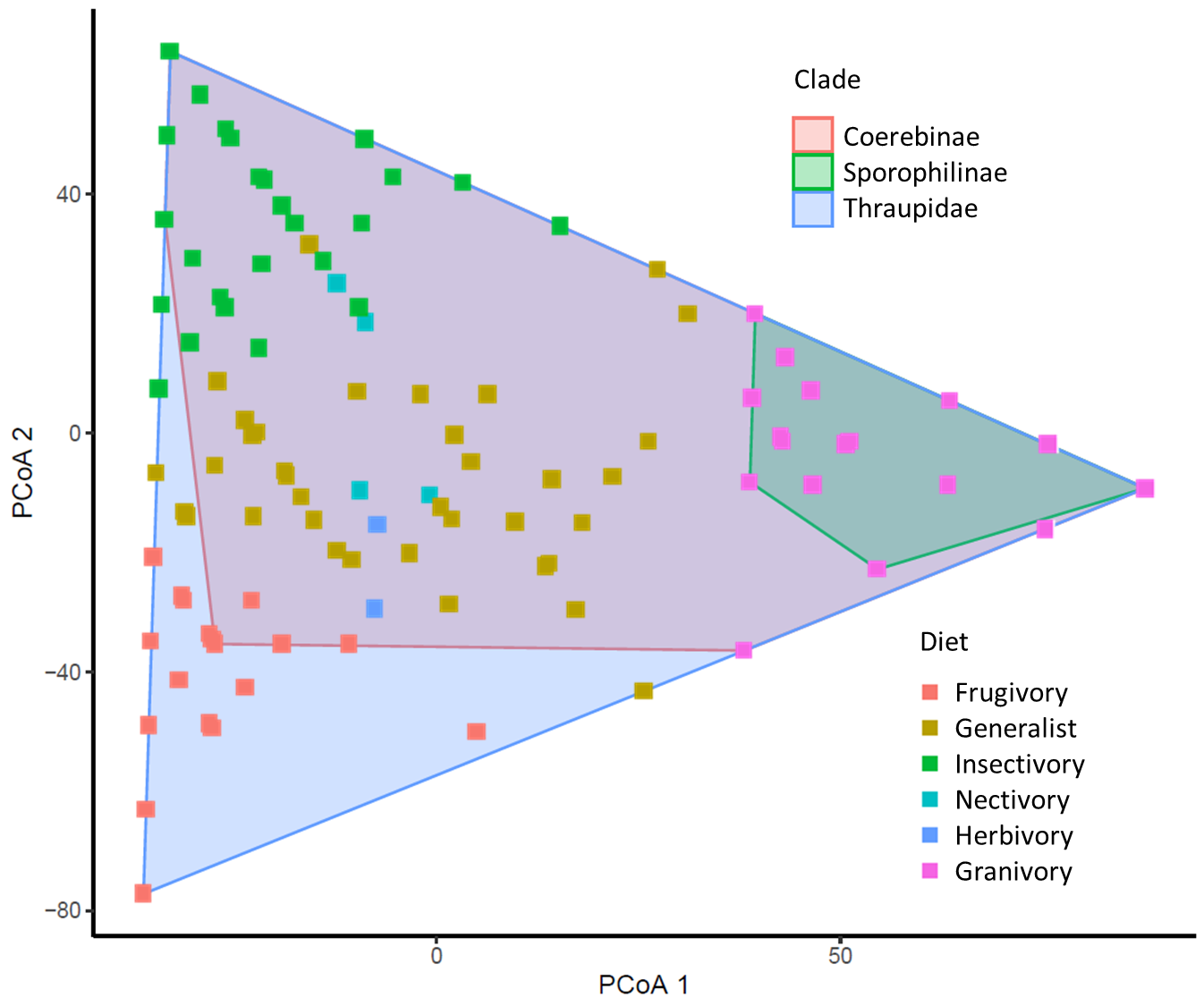


**Figure 25** Contributions of beak traits to individual principal component axes in figure 4a. Red dashed line on each bar chart is the null expectation (25%) of each trait contribution to a component axis.


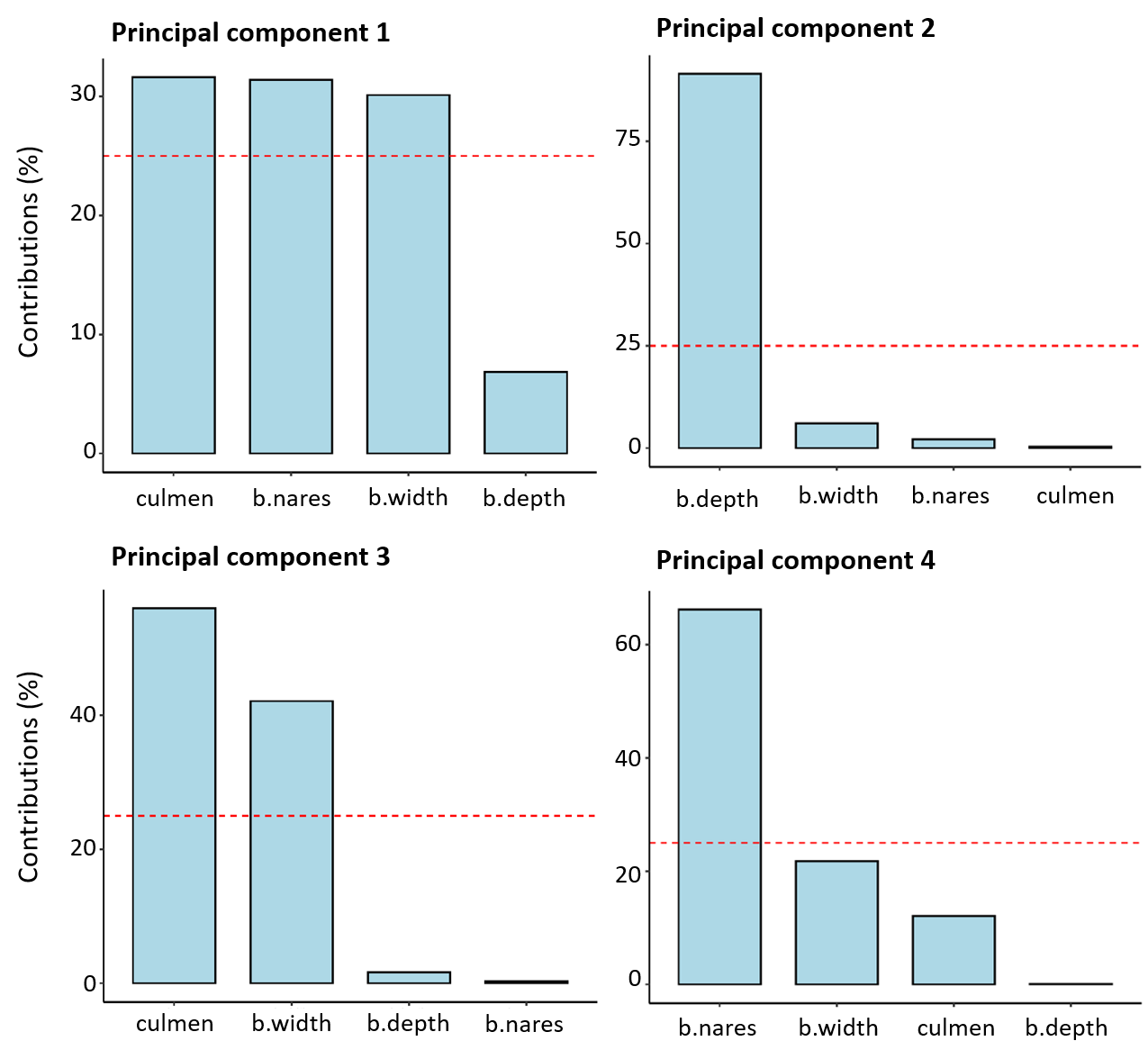


**Figure 26** Contributions of beak traits to individual principal component axes in figure 5. Red dashed line on each bar chart is the null expectation (25%) of each trait contribution to a component axis.


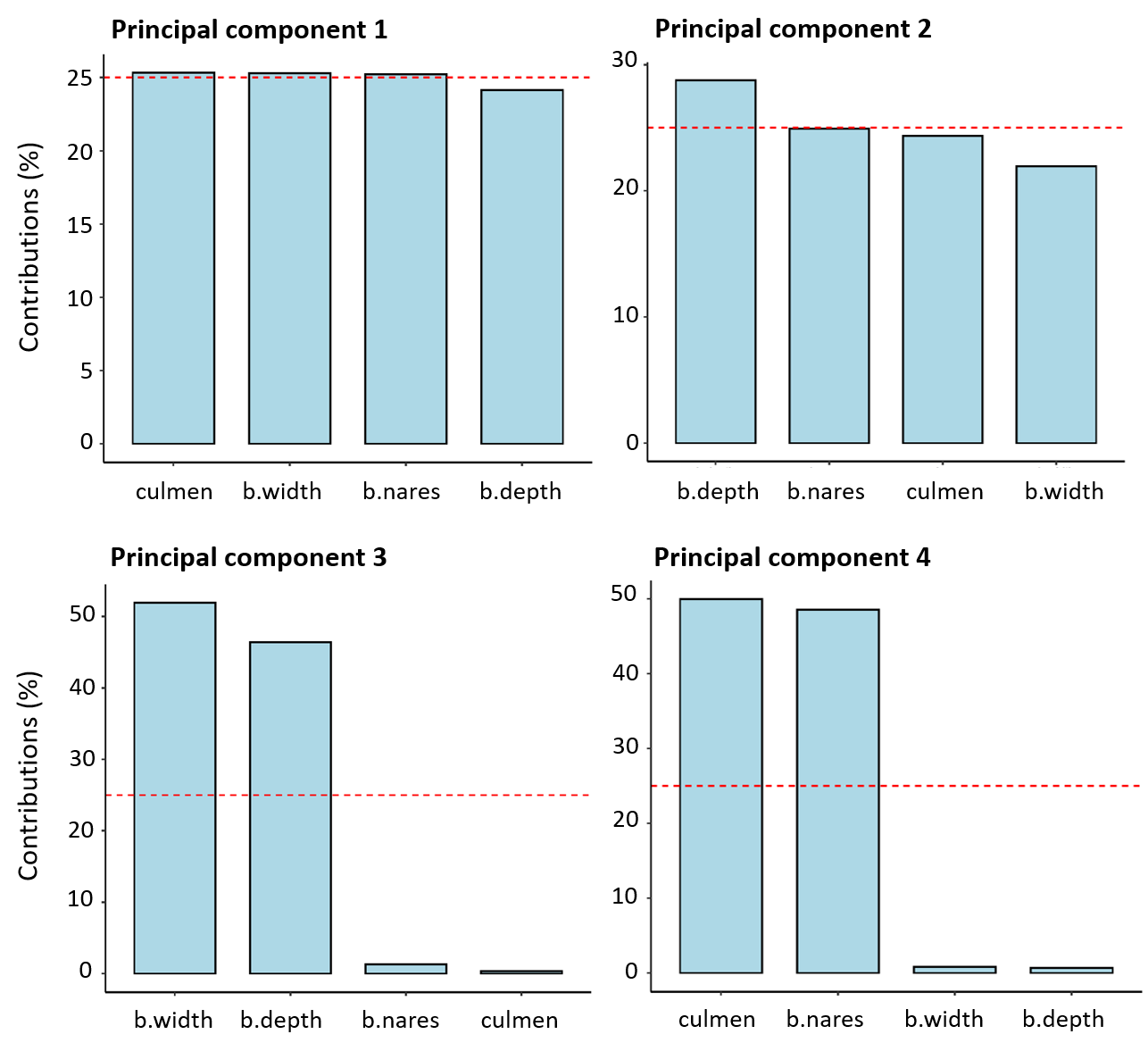


**Table 1** Posterior probabilities of nodes among the Coerebinae branches of the MCC tree. Node support values are taken from the BEAST analysis from Burns et al. (2014).

| **Species** | **Node support** | | | | | | | | | | | | | | | |
| --- | --- | --- | --- | --- | --- | --- | --- | --- | --- | --- | --- | --- | --- | --- | --- | --- |
| Coereba flaveola |  |  |  |  |  |  |  |  |  |  |  |  |  |  |  | 1.00 |
| Tiaris olivaceus |  |  |  |  |  |  |  |  |  |  |  |  |  |  | 0.99 |  |
| Euneornis campestris |  |  | 0.35 |  |  |  |  |  |  |  |  |  |  | 1.00 |  |  |
| Loxigilla portoricensis |  | 1.00 |  |  |  |  |  |  |  |  |  |  |  |  |  |  |
| Melopyrrha nigra | 0.93 |  |  |  |  |  |  |  |  |  |  |  |  |  |  |  |
| Loxigilla violacea |  |  |  |  |  |  |  |  |  |  |  |  |  |  |  |  |
| Loxipasser anoxanthus |  |  |  |  |  |  |  |  |  |  |  |  | 1.00 |  |  |  |
| Tiaris canorus |  |  |  |  |  |  |  |  |  |  |  | 0.74 |  |  |  |  |
| Loxigilla noctis | 1.00 |  |  |  |  |  |  |  |  |  | 0.61 |  |  |  |  |  |
| Loxigilla barbadensis |  |  |  |  |  |  |  |  |  |  |  |  |  |  |  |  |
| Tiaris bicolor | 1.00 |  |  |  |  |  |  |  |  | 0.68 |  |  |  |  |  |  |
| Melanospiza richardsoni |  |  |  |  |  |  |  |  |  |  |  |  |  |  |  |  |
| Tiaris obscurus | 1.00 |  |  |  |  |  |  |  | 1.00 |  |  |  |  |  |  |  |
| Tiaris fuliginosus |  |  |  |  |  |  |  |  |  |  |  |  |  |  |  |  |
| Certhidea olivacea |  |  |  |  |  |  |  | 0.88 |  |  |  |  |  |  |  |  |
| Platyspiza crassirostris |  |  |  |  |  |  | 0.97 |  |  |  |  |  |  |  |  |  |
| Pinaroloxias inornata |  |  |  |  |  | 0.99 |  |  |  |  |  |  |  |  |  |  |
| Camarhynchus heliobates |  |  |  | 0.71 | 0.66 |  |  |  |  |  |  |  |  |  |  |  |
| Camarhynchus pauper |  |  | 0.94 |  |  |  |  |  |  |  |  |  |  |  |  |  |
| Camarhynchus pallidus |  | 0.22 |  |  |  |  |  |  |  |  |  |  |  |  |  |  |
| Camarhynchus psittacula | 0.41 |  |  |  |  |  |  |  |  |  |  |  |  |  |  |  |
| Camarhynchus parvulus |  |  |  |  |  |  |  |  |  |  |  |  |  |  |  |  |
| Geospiza fuliginosa |  |  |  | 0.50 |  |  |  |  |  |  |  |  |  |  |  |  |
| Geospiza difficilis |  |  | 0.93 |  |  |  |  |  |  |  |  |  |  |  |  |  |
| Geospiza fortis | 0.14 | 0.52 |  |  |  |  |  |  |  |  |  |  |  |  |  |  |
| Geospiza scandens |  |  |  |  |  |  |  |  |  |  |  |  |  |  |  |  |
| Geospiza magnirostris | 0.40 |  |  |  |  |  |  |  |  |  |  |  |  |  |  |  |
| Geospiza conirostris |  |  |  |  |  |  |  |  |  |  |  |  |  |  |  |  |

**Table 2** Posterior probabilities of nodes among the Sporophilinae branches of the MCC tree. Node support values are taken from the BEAST analysis from Burns et al. (2014).

| **Species** | **Node support** | | | | | |
| --- | --- | --- | --- | --- | --- | --- |
| Sporophila lineola |  |  |  |  |  | 1 |
| Oryzoborus crassirostris | 1 | 1 |  |  |  |  |
| Oryzoborus atrirostris |  |  |  |  |  |  |
| Oryzoborus maximiliani | 1 |  |  |  |  |  |
| Oryzoborus nuttingi |  |  |  |  |  |  |
| Sporophila leucoptera |  |  | 1 |  | 1 |  |
| Sporophila peruviana |  | 1 |  |  |  |  |
| Sporophila simplex | 1 |  |  |  |  |  |
| Sporophila telasco |  |  |  |  |  |  |
| Sporophila castaneiventris |  |  |  | 1 |  |  |
| Sporophila minuta |  |  | 1 |  |  |  |
| Sporophila melanogaster | 0.44 | 1 |  |  |  |  |
| Sporophila cinnamomea |  |  |  |  |  |  |
| Sporophila pileata | 0.93 |  |  |  |  |  |
| Sporophila hypochroma |  |  |  |  |  |  |
| Sporophila intermedia | 0.82 |  |  |  | 0.76 |  |
| Sporophila corvina |  |  |  |  |  |  |
| Sporophila schistacea | 1 |  | 1 |  |  |  |
| Sporophila falcirostris |  |  |  |  |  |  |
| Sporophila plumbea |  | 1 |  |  |  |  |
| Sporophila albogularis | 1 |  |  |  |  |  |
| Sporophila collaris |  |  |  |  |  |  |
| Sporophila torqueola |  | 0.88 |  | 0.22 |  |  |
| Oryzoborus funereus | 1 |  |  |  |  |  |
| Oryzoborus angolensis |  |  |  |  |  |  |
| Dolospingus fringilloides | 0.43 |  | 0.71 |  |  |  |
| Sporophila frontalis |  |  |  |  |  |  |
| Sporophila luctuosa |  | 0.96 |  |  |  |  |
| Sporophila caerulescens | 0.99 |  |  |  |  |  |
| Sporophila nigricollis |  |  |  |  |  |  |

**Table 3** Posterior probabilities of the specified shifts in morphological evolution identified in bayou. The order of shifts goes from top to bottom as viewed in appendix figures 14 to 23 and do not include the background regime (shown in red). Abbreviated trait names are Bnares (beak tip to nares length). Values in bold are shifts present along branches of Coerebinae (^+^) and Sporophilinae (^o^) taxa.

| **Shift** | **Body Mass** | **Culmen length** | **Beak nares** | **Beak width** | **Beak depth** | **Tarsus length** | **Wing length** | **Hand-wing index** | **Tail length** |
| --- | --- | --- | --- | --- | --- | --- | --- | --- | --- |
| 1 | 0.822 | 0.890 | 0.998 | 0.540 | 0.651 | 0.553 | 0.250 | 0.999 | 0.405 |
| 2 | 0.893 | 0.728 | 0.512 | 0.729 | 0.995 |  | 0.252 | 0.986 |  |
| 3 | 0.932 | 0.779 | 0.507 | 0.843 | 0.646 |  |  | 0.997 |  |
| 4 | 0.904 | **0.607 ^(o)^** | 0.851 | 0.998 | 0.995 |  |  | 0.986 |  |
| 5 | 0.639 |  | 0.548 | 0.940 | 0.927 |  |  | 0.541 |  |
| 6 | 0.776 |  | 0.556 | 1.000 | 0.812 |  |  | 0.747 |  |
| 7 | 0.589 |  | 0.523 | 0.996 | **0.812 ^(+)^** |  |  | 0.952 |  |
| 8 | 0.502 |  |  | 0.627 | **0.669 ^(+)^** |  |  | 0.863 |  |
| 9 | 0.865 |  |  | **0.750 ^(+)^** | **0.994 ^(o)^** |  |  | 0.972 |  |
| 10 | 0.677 |  |  | **0.999 ^(o)^** | 0.999 |  |  |  |  |
| 11 | 0.797 |  |  | 0.880 | 0.855 |  |  |  |  |
| 12 |  |  |  | 0.668 |  |  |  |  |  |

**Table 4** Parameter values of bayou output of Thraupidae tree. Shifts indicate number of regimes shifts across the tree for various traits with a posterior probability greater than 0.5.

| **Variable** | | **Body Mass** | **Culmen length** | **Beak nares** | **Beak width** | **Beak depth** | **Tarsus length** | **Wing length** | **Hand-wing index** | **Tail length** |  |
| --- | --- | --- | --- | --- | --- | --- | --- | --- | --- | --- | --- |
| No. of shifts | 11 | | 4 | 7 | 12 | 11 | 1 | 0 | 9 | 0 |  |
| LogLik | -106.26 | | 137.9 | 120.15 | 142.2 | -593.35 | 233.5 | 227.1 | 184.75 | 149.25 |  |
| Half-life | 1 | | 2.08 | 3.49 | 0.67 | 0.15 | 29.24 | 15.67 | 0.18 | 67.56 | |

**Table 5** Comparisons of model fit for Thraupidae morphological traits. Thetas values don’t define an evolutionary regime. Applying BM models across the phylogeny using stochastic evolution recognises 68 regimes or measurements that differ significantly between lineages.

| **Body Mass** |  | |  | |  | |  | |  | |  | |  | |
| --- | --- | --- | --- | --- | --- | --- | --- | --- | --- | --- | --- | --- | --- | --- |
| Variable | BM1 | | BMS | | OU1 | | OUM | | OUMV | | OUMA | | OUMVA | |
| No. of shifts | 18 | | 18 | | 18 | | 18 | | 18 | | 18 | | 18 | |
| LogLik | -1364.07 | | -1320.04 | | -1348.39 | | -1326.53 | | -1276.84 | | -1260.08 | | 1397.49 | |
| Half-life | - | | - | | 26.35 | | 0.05 | | 11.14 | | 5.05 | | 2.45 | |
| AICc | 2732.17 | | 2720.51 | | 2702.84 | | 2695.58 | | 2636.61 | | 2603.09 | | -2667.05 | |
| param.count | 2 | | 36 | | 3 | | 20 | | 37 | | 37 | | 54 | |
|  |  | |  | |  | |  | |  | |  | |  | |
| **Beak Tip to Nares Length** | | | |  | |  | |  | |  | |  | |  |
| Variable | BM1 | | BMS | | OU1 | | OUM | | OUMV | | OUMA | | OUMVA | |
| No. of shifts | 8 | | 8 | | 8 | | 8 | | 8 | | 8 | | 8 | |
| LogLik | -823.93 | | -763.45 | | -764.56 | | -701.34 | | -675.53 | | 5764.29 | | 2.05E+09 | |
| Half-life | - | | - | | 10.95 | | 2.66 | | 2.74 | | 1.21 | | 5.61 | |
| AICc | 1651.89 | | 1560.52 | | 1535.18 | | 1423.33 | | 1386.88 | | -11492.8 | | -4.09E+09 | |
| param.count | 2 | | 16 | | 3 | | 10 | | 17 | | 17 | | 24 | |
|  |  | |  | |  | |  | |  | |  | |  | |
| **Beak Width** | |  | |  | |  | |  | |  | |  | |  |
| Variable | BM1 | | BMS | | OU1 | | OUM | | OUMV | | OUMA | | OUMVA | |
| No. of shifts | 20 | | 20 | | 20 | | 20 | | 20 | | 20 | | 20 | |
| LogLik | -690.14 | | -527.88 | | -631.63 | | -511.47 | | -398.62 | | 11147.54 | | 12213.43 | |
| Half-life | - | | - | | 12.29 | | 0.09 | | 0.09 | | 0.11 | | 0.11 | |
| AICc | 1384.31 | | 1146.28 | | 1269.33 | | 1070 | | 890.31 | | -22202 | | -24281.78 | |
| param.count | 2 | | 40 | | 3 | | 22 | | 41 | | 41 | | 60 | |
|  |  | |  | |  | |  | |  | |  | |  | |
| **Beak Depth** | |  | |  | |  | |  | |  | |  | |  |
| Variable | BM1 | | BMS | | OU1 | | OUM | | OUMV | | OUMA | | OUMVA | |
| No. of shifts | 14 | | 14 | | 14 | | 14 | | 14 | | 14 | | 14 | |
| LogLik | -845.80 | | -630.60 | | -780.40 | | -622.90 | | -552.16 | | 8465.91 | | 17166.22 | |
| Half-life | - | | - | | 10.87 | | 0.42 | | 0.42 | | 0.33 | | 0.32 | |
| AICc | 1695.71 | | 1322.34 | | 1566.9 | | 1279.44 | | 1167.71 | | -16868.4 | | -34236.78 | |
| param.count | 2 | | 28 | | 3 | | 16 | | 29 | | 29 | | 42 | |
|  |  | |  | |  | |  | |  | |  | |  | |

| **Tarsus Length** | |  | |  | |  | |  | |  | |  | | |
| --- | --- | --- | --- | --- | --- | --- | --- | --- | --- | --- | --- | --- | --- | --- |
| Variable | BM1 | | BMS | | OU1 | | OUM | | OUMV | | OUMA | | OUMVA | |
| No. of shifts | 8 | | 8 | | 8 | | 8 | | 8 | | 8 | | 8 | |
| LogLik | -858.35 | | -812.1 | | -848.26 | | -813.15 | | -794.47 | | -794.44 | | 2544.63 | |
| Half-life | - | | - | | 34.53 | | 15.81 | | 19.15 | | 13.65 | | 0.65 | |
| AICc | 1720.73 | | 1657.82 | | 1702.59 | | 1646.94 | | 1624.77 | | 1624.72 | | -5037.61 | |
| param.count | 2 | | 16 | | 3 | | 10 | | 17 | | 17 | | 24 | |
|  |  | |  | |  | |  | |  | |  | |  | |
| **Wing chord Length** | |  | |  | |  | |  | |  | |  | |  |
| Variable | BM1 | | BMS | | OU1 | | OUM | | OUMV | | OUMA | | OUMVA | |
| No. of shifts | 10 | | 10 | | 10 | | 10 | | 10 | | 10 | | 10 | |
| LogLik | -1325.03 | | -1287.92 | | -1317.62 | | -1297.65 | | -1274.02 | | 6239.28 | | 7707.08 | |
| Half-life | - | | - | | 39.58 | | 24.99 | | 25.34 | | 1.65 | | 3.15 | |
| AICc | 2654.09 | | 2618.36 | | 2641.3 | | 2620.22 | | 2592.83 | | -12433.8 | | -15348.38 | |
| param.count | 2 | | 20 | | 3 | | 12 | | 21 | | 21 | | 30 | |
|  |  | |  | |  | |  | |  | |  | |  | |
| **Hand-wing Index** | |  | |  | |  | |  | |  | |  | |  |
| Variable | BM1 | | BMS | | OU1 | | OUM | | OUMV | | OUMA | | OUMVA | |
| No. of shifts | 12 | | 12 | | 12 | | 12 | | 12 | | 12 | | 12 | |
| LogLik | -1044.19 | | -991.83 | | -989.7 | | -870.99 | | -849.19 | | 7936.03 | | 1.17E+15 | |
| Half-life | - | | - | | 11.87 | | 0.19 | | 0.17 | | 0.45 | | 0.19 | |
| AICc | 2092.41 | | 2035.31 | | 1985.46 | | 1771.23 | | 1752.35 | | -15818.1 | | -2.33E+15 | |
| param.count | 2 | | 24 | | 3 | | 14 | | 25 | | 25 | | 36 | |
|  |  | |  | |  | |  | |  | |  | |  | |
| **Tail Length** |  |  | |  | |  | |  | |  | |  | |  |
| Variable | BM1 | | BMS | | OU1 | | OUM | | OUMV | | OUMA | | OUMVA | |
| No. of shifts | 6 | | 6 | | 6 | | 6 | | 6 | | 6 | | 6 | |
| LogLik | -1293.22 | | -1283.83 | | -1293.09 | | -1286.01 | | -1282.4 | | 3457.69 | | 3664.63 | |
| Half-life | - | | - | | 323.89 | | 99.77 | | 89.24 | | 2.54 | | 1.17 | |
| AICc | 2590.48 | | 2592.57 | | 2592.26 | | 2588.44 | | 2591.88 | | -6888.3 | | -7291.22 | |
| param.count | 2 | | 12 | | 3 | | 8 | | 13 | | 3 | | 18 | |
|  |  | |  | |  | |  | |  | |  | |  | |

**Table 6** GEE output relating diet type with changes in beak dimensions. Estimate values of beak dimensions indicate that a single unit increase in diet type (the response variable) is associated with an increase/decrease in a particular beak dimension (the predictor). S.E. is the standard error and significant p values are denoted with asterisks; p<0.05 (*), p<0.01 (**) and p<0.001 (***)

| **Invertebrate** | Estimate | S.E. | t | p value |
| --- | --- | --- | --- | --- |
| (Intercept) | -0.929 | 0.175 | -5.321 | <0.001 *** |
| culmen | 0.881 | 0.094 | 9.39 | <0.001 *** |
| beak nares | -0.213 | 0.085 | -2.505 | 0.014 |
| beak width | -0.002 | 0.077 | -0.032 | 0.975 |
| beak depth | -0.405 | 0.066 | -6.143 | <0.001 *** |
|  |  |  |  |  |
| **Fruit** | Estimate | S.E. | t | p value |
| (Intercept) | 1.104 | 0.217 | 5.081 | <0.001 *** |
| culmen | -1.215 | 0.117 | -10.402 | <0.001 *** |
| beak nares | 0.726 | 0.106 | 6.854 | <0.001 *** |
| beak width | 0.26 | 0.096 | 2.7 | 0.008 ** |
| beak depth | 0.275 | 0.082 | 3.355 | <0.001 *** |
|  |  |  |  |  |
| **Nectar** | Estimate | S.E. | t | p value |
| (Intercept) | 0.436 | 0.091 | 4.797 | <0.001 *** |
| culmen | 0.083 | 0.049 | 1.689 | 0.094 |
| beak nares | -0.132 | 0.044 | -2.971 | 0.004 ** |
| beak width | 0.223 | 0.04 | 5.529 | <0.001 *** |
| beak depth | -0.394 | 0.034 | -11.484 | <0.001 *** |
|  |  |  |  |  |
| **Seed** | Estimate | S.E. | t | p value |
| (Intercept) | 1.728 | 0.197 | 8.78 | <0.001 *** |
| culmen | -0.907 | 0.106 | -8.567 | <0.001 *** |
| beak nares | 0.678 | 0.096 | 7.06 | <0.001 *** |
| beak width | -0.701 | 0.087 | -8.021 | <0.001 *** |
| beak depth | 0.361 | 0.074 | 4.868 | <0.001 *** |
|  |  |  |  |  |
| **Plant** | Estimate | S.E. | t | p value |
| (Intercept) | 0.205 | 0.042 | 4.891 | <0.001 *** |
| culmen | -0.068 | 0.023 | -3.005 | 0.003 * |
| beak nares | -0.144 | 0.02 | -7.024 | <0.001 *** |
| beak width | -0.267 | 0.019 | -14.368 | <0.001 *** |
| beak depth | 0.397 | 0.016 | 25.098 | <0.001 *** |
